# Impact of a Contextualized Workplace Intervention in a Latino Population on Reducing Cardiovascular Risk and Its Associated Factors

**DOI:** 10.3390/jcm15020628

**Published:** 2026-01-13

**Authors:** Yoredy Sarmiento-Andrade, María Alejandra Ojeda Ordóñez, Juan Pablo Sisalima, Rosario Suárez, Rowland Snell Astudillo Cabrera, Estefanía Bautista-Valarezo, Bárbara Badanta

**Affiliations:** 1 Facultad de Ciencias de la Salud, Universidad Técnica Particular de Loja, Loja 110150, Ecuador; maojeda@utpl.edu.ec (M.A.O.O.); jpsisalima@utpl.edu.ec (J.P.S.); rsuarez2@utpl.edu.ec (R.S.); mebautista@utpl.edu.ec (E.B.-V.); 2 Centro Clínico Quirúrgico-Hospital del día del Instituto Ecuatoriano de Seguridad Social (IESS), Loja 110150, Ecuador; drastudilloc@gmail.com; 3 Department of Nursing, University of Seville, 41009 Sevilla, Spain; bbadanta@us.es

**Keywords:** cardiovascular risk, early medical intervention, healthy lifestyle, risk factors, health education

## Abstract

**Background:** Cardiovascular diseases (CVD) are the leading global cause of death, disproportionately affecting Latin America. This study evaluated the impact of a contextualized workplace intervention, adapted from the Diabetes Prevention Program (DPP), on reducing cardiovascular risk (CVR) in a Latin American population. **Methods:** A quasi-experimental, pre-post study was conducted with 100 adults (34 males, 66 females) affiliated with the social security system. The 16-week “Transforma tu vida con cambios diarios” program, included ten sessions focused on motivation, healthy eating and physical activity. Sociodemographic, anthropometric, clinical, and biochemical parameters were measured before and after the intervention. CVR was estimated as a 10-year risk percentage using the non-laboratory Globorisk model. Analysis included paired *t*-test and Cohen’s *d* effect sizes. **Results:** Significant improvements (*p* < 0.05) were associated with the intervention. The predicted mean CVR score decreased from 8.03% to 6.71% (*p* = 0.03, *d* = 0.658). Reductions were observed in weight (73.1 to 71.7 kg, *p* < 0.001, *d* = 0.424), BMI (29.0 to 28.5 kg/m^2^, *p* < 0.001, *d* = 0.363), and physical inactivity (60% to 39%, *p* = 0.001). A moderate-low clinical impact was found for systolic blood pressure (124.9 to 121.2 mmHg; *p* = 0.003, *d* = 0.301) and glucose (103.3 to 101.1 mg/dL; *p* = 0.04, *d* = 0.218) and HDL cholesterol (51.5 to 54.9 mg/dL; *p* = 0.02, *d* = −0.286) showed significant but small effects. **Conclusions:** The intervention was associated with favorable changes in clinical and anthropometric indicators. The results provide preliminary evidence that logistical adaptation to the workplace can effectively reach at-risk Latino populations, with weight and BMI improvements reflecting the program’s strong physical activity component.

## 1. Introduction

Cardiovascular diseases (CVD) are the leading cause of death globally and are responsible for approximately 17.9 million fatalities annually according to the World Health Organization. In the Americas, CVD accounts for 75% of all deaths [1,2] with the burden particularly pronounced in low- and middle-income countries, where social, cultural, economic, and lifestyle inequalities increase cardiovascular risk (CVR) [3,4]. In this regard, Latin America faces social, economic, and cultural disparities that hinder the prevention of chronic diseases such as CVD and T2D [5].

In Ecuador, CVD causes about 25.7% of all deaths [6], constituting the primary reason for medical consultation. The STEPS survey which is the progressive stepwise study of risk factors for chronic non-communicable diseases, revealed a critical diagnostic and control gap: that 45.2% of hypertensive Ecuadorians were unaware of their condition and only 26% achieved effective pharmacological control; additionally, 50.1% had never undergone glucose or lipid testing, both critical markers of CVR [7,8] Ecuador is no exception; its cultural diversity complicates public health interventions and efforts to reduce risk factors [9]. This reality suggests that traditional clinical interventions are insufficient if social determinants and access to primary prevention are not addressed in the settings where the population spends most of its time.

The Diabetes Prevention Program (DPP) of the U.S. Centers for Disease Control and Prevention (CDC) represents a gold standard for lifestyle modification interventions [4,10,11]. However, most of the evidence on its effectiveness comes from controlled clinical trials in high-income countries, with diets and social structures that are foreign to the Andean reality. There is an urgent need to assess whether this model remains effective when shifted from a purely diabetogenic approach to a comprehensive cardiovascular risk approach, and from a clinical setting to a workplace setting.

Several studies have shown that workplace-based lifestyle interventions have emerged as promising strategies [8,12,13]. However, in Latin America, these initiatives tend to be sporadic and lack a structured methodology based on validated programmes such as the DPP. This study differs from previous research in that it proposes “deep cultural contextualisation in the workplace”: not only adapting nutritional content to the Ecuadorian basic food basket but also integrating hybrid modalities (face-to-face and virtual) that respond to current workplace dynamics.

We justify this research on the premise that the work environment, mediated by the Instituto Ecuatoriano de Seguridad Social (IESS), the main healthcare provider for formal workers, provides an appropriate infrastructure for preventive medicine, making it an ideal institution for structured interventions with a significant impact. Therefore, the objective of this study was to evaluate the effectiveness of the program “Transforma tu vida con cambios diarios”, an adaptation based on the DPP, in adults with cardiovascular risk in the Ecuadorian workplace. The results seek to demonstrate that a scalable program culturally adapted to the workplace can reduce CVR scores in a middle-income Latin American population, closing the gap between international guidelines and local practice and highlighting the need to integrate lifestyle programs into public health systems, thereby improving primary prevention of CVD in the region.

## 2. Materials and Methods

### 2.1. Study Design and Participants

This quasi-experimental pre-post intervention design. The source population consisted of 1406 employees from public and private institutions affiliated with the IESS, in southern Ecuador, between November 2023 and February 2024. The study was conducted in accordance with JBI checklist for quasi-experimental studies 2023 [14]

Access to the affiliated population was facilitated through a formal institutional collaboration agreement between the research team and the healthcare provider, enabling systematic identification and recruitment of potential participants. Eligibility was assessed using the inclusion criteria established in the Globorisk cardiovascular risk prediction model, which included age between 40 and 75 years and classification of 10-year cardiovascular risk as low (<5%), moderate (5–9%), high (10–19%) or very high (≥20%). Based on these criteria, 357 individuals were identified as eligible [15].

**Figure 1:**
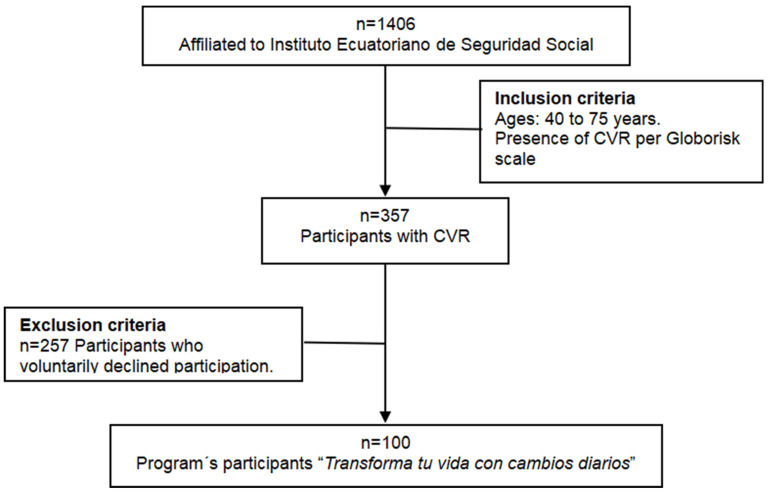
Flowchart of the recruitment process of participants in the intervention study.

A non-probabilistic sampling approach was applied. Of the eligible individuals, 100 participants voluntarily agreed to enroll in the lifestyle intervention program “Transforma tu vida con cambios diarios” No participants attrition occurred during f the intervention period, and all enrolled individuals completed both baseline and post-intervention assessments conducted four months after program initiation. The remaining 257 eligible individuals declined participation. The participant recruitment process is illustrated in Figure 1.

### 2.2. Intervention Strategy

**Figure 2:**
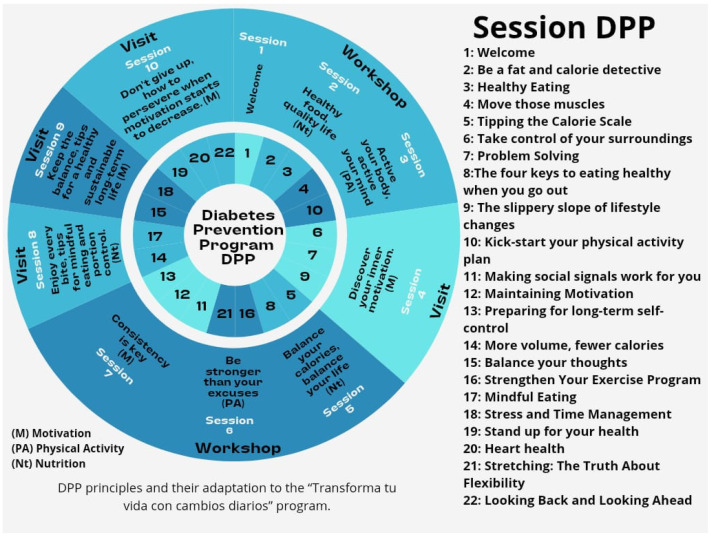
Adaptation of the DPP to the “Transforma tu vida con cambios diarios” program.

The programme “Transforma tu vida con cambios diarios” consisted of an adaptation of the original DPP curriculum. It is important to highlight that the DPP curriculum is a public domain resource designed for global replication and adaptation; therefore, this intervention adjusted the original guidelines to the socio-cultural realities and labor conditions of the Ecuadorian participants (Figure 2). The intervention was structured into 10 sessions organized around three fundamental pillars: motivation, healthy eating, and physical activity.

To ensure transparency and facilitate the study’s replicability, a detailed description of the intervention is included as Supplementary Materials (Table S1), describing the topics and activities implemented in each session. The activities included:

#### 2.2.1. Face-to-Face Theoretical-Practical Workshops

Two educational and participatory workshops were held, with simultaneous and rotating stations every 45 min.

##### Workshop 1 (Sessions 1, 2 and 3)

This included a welcome session, a nutrition session focused on education on fats and macronutrients, and an introduction to physical activity session through simple routines, self-assessment of physical condition and weekly exercise planning.

##### Workshop 2 (Sessions 5, 6 and 7)

This workshop explored energy balance, the progression of physical exercise to reduce sedentary behavior, and behavioral adherence strategies, incorporating barrier resolution and stress management dynamics.

#### 2.2.2. Group Follow-Up Visits

Four intermediate visits (sessions 4, 8, 9, and 10) were made to the participants’ workplaces, with the number of participants varying according to the total number of people enrolled in the specific institution visited each day. These visits included weight control, personalized feedback, goal review, nutritional advice with food models, and brief motivational talks. The visits were adapted to the work schedule, ensuring participation without interfering with the workday. Participants recorded their achievements in a standardized printed booklet (Booklet S1), prepared and reviewed in advance by the researchers, which were given to each participant at the first workshop.

#### 2.2.3. Asynchronous Digital Support

Channels managed by the research team were designed to reinforce the content of the face-to-face sessions, acting as a means of educational and behavioral reinforcement, promoting sustained engagement. Participation was established through the receipt and viewing of the material by the participants.

##### WhatsApp Group (Non-Interactive)

A one-way communication tool through which researchers sent reminders of session dates and times, digital educational material such as short explanatory videos, key slides from face-to-face sessions, and motivational messages based on the topics of the adapted sessions.

##### Instagram Account

Used exclusively as a visual repository and easy access to educational material. Its content was aligned with the key topics of the intervention sessions (stress management techniques, nutritional information, and strategies for creating planned physical activity habits).

The non-interactive nature of these channels was a methodological decision to maintain the homogeneity of the intervention among participants, ensure privacy, and avoid informal interactions. Any questions from participants were channeled through direct telephone contact with the research team.

### 2.3. Monitoring and Adherence

**Figure 3:**
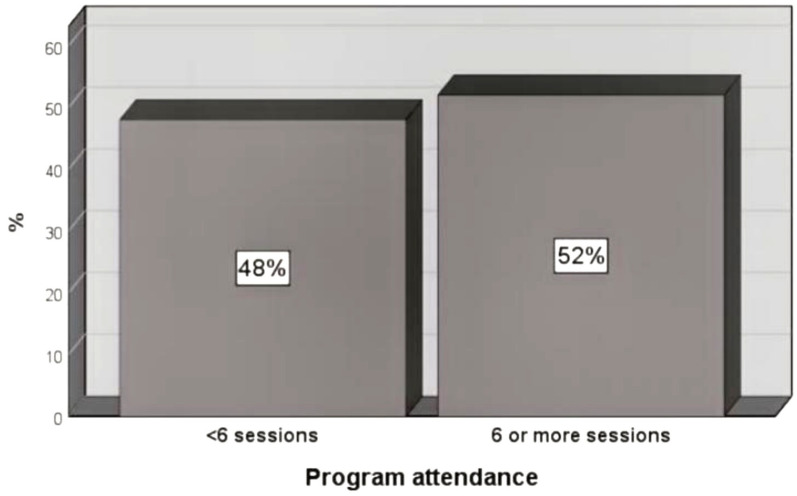
This figure shows that the majority (52%) of individuals attained satisfactory attendance (six or more sessions), according to the data, while 48% of participants had unsatisfactory attendance (less than six sessions).

Attendance was recorded at each face-to-face session and follow-up visit. In addition, researchers verified weekly that self-assessment brochures had been completed. Minimum participation of 60% of sessions was required to be considered adherent to the program (Figure 3).

### 2.4. Data Collection

Data was collected at baseline (T1) and post-intervention (T2) folowing STEPwise method 3.2 standards, adapted to Ecuador by Ministerio de Salud Pública (MSP) with INEC and PAHO/WHO [16]. Information included sociodemographic variables (age, gender, education level, marital status, and income categorized according to Ecuador’s 2024 Unified Basic Salary of $460 USD) [17], plus clinical, anthropometric, and biochemical data. The dependent variable, CVR, was assessed using the Globorisk scale.

#### 2.4.1. Clinical and Behavioral Assessments

Cardiovascular risk was estimated using the non-laboratory-based version of the Globorisk prediction model, that has risk charts for 182 countries [18]. This office-based (non-laboratory) version of the Globorisk prediction model estimates the 10-year risk percentage of both fatal and non-fatal cardiovascular disease. This model uses five predictor variables—age, sex, smoking status, systolic blood pressure (SBP), and body mass index (BMI). It is acknowledged that changes in this score are mathematically driven by modifications in its input variables (weight, BP, etc.). This model uses BMI as a verified stand-in for metabolic markers, including total cholesterol and glucose levels [19]. Weekly physical activity was measured using the International Physical Activity Questionnaire (IPAQ), a validated tool for moderate to vigorous activity, sedentary behavior [16]; and tobacco use was also assessed.

Blood pressure was measured under American Heart Association guidelines [20,21], using the OMRON HEM-7120 Arm Monitor, accurate to ±3 mmHg [22] and validated by the International Protocol of the European Society of Hypertension.

#### 2.4.2. Body Composition

Body composition was assessed with the InBody 120^®^ bioelectrical impedance analyzer, a non-invasive tool strongly correlated with anthropometric methods and computed tomography, particularly for visceral fat [23]. Measurements included weight, body mass index (BMI), waist-hip ratio (WHR), muscle mass, body fat percentage, fat mass, and visceral fat. Waist circumference was measured at the umbilical level at the end of expiration with a Cescorf tape, resolution ± 1 mm.

#### 2.4.3. Laboratory Parameters

Fasting blood glucose, a key marker associated with CVR [24], was assessed using the Blood Glucose Monitor model SD CHECK^®^ GOLD (manufactured by SD BIOSENSOR, INC. City: Suwon-si (Gyeonggi-do Province). Country of origin: South Korea (Republic of Korea)), providing reliable results in 5 s through high-conductivity electrodes. Capillary fasting blood samples (0.9 µL) were collected fasting lancets and pipettes.

Lipid profile, an indicator for early detection of metabolic alterations and CVR, was measured using the STANDARD LipidoCare^®^ Analyzer (manufactured by SD BIOSENSOR, INC. City: Suwon-si (Gyeonggi-do Province). Country of origin: South Korea (Republic of Korea)), which evaluates cholesterol and triglycerides from fasting capillary blood. This method has high sensitivity and specificity, supporting early detection of lipid disorders [25].

Quality control of biochemical measurements was performed using standardized portable capillary analysis equipment as described previously. To ensure fasting metabolic status, participants were instructed to fast for at least 10 h prior to sampling. Before blood collection, fasting compliance was verified verbally, and sampling was scheduled between 7:00 and 9:00 a.m.

### 2.5. Statistical Analysis

Data were analyzed using SPSS version 26. Quantitative variables were presented with central tendency and dispersion after normality testing. Qualitative variables were summarized through absolute and relative frequency distributions

For bivariate analysis, chi-square tests and independent samples *t*-tests were applied. Comparative analyses before and after the intervention used McNemar’s test for categorical variables and paired *t*-tests for continuous variables. For continuous variables, the pre-post mean differences were computed as Pre–Post. As a result, negative values indicate a numerical rise and positive values indicate a decrease in the parameter Sex-stratified analyses were performed to explore differential intervention effects in men and women

Given the observed changes in diastolic blood pressure (DBP), an additional (pre-post) stratified analysis was conducted: the sample was divided into two subgroups according to baseline DBP status (<90 mmHg vs. ≥90 mmHg) because to the notable increase in the mean DBP of the entire cohort. To ascertain whether the mean increase was caused by a detrimental effect or by physiological normalization of low levels, paired *t*-tests were conducted independently on these subgroups.

Effect sizes were calculated using Cohen’s *d* to quantify the magnitude of pre-post changes. Effect sizes were interpreted as low (0.2), moderate (0.5), or large (≥0.8). For effect size estimates, 95% confidence intervals (CI) were reported. Statitical significane was defined as *p* < 0.05.

### 2.6. Ethical Considerations

The protocol for this study was approved by the Human Research Ethics Committee (CEISH) of The Hospital General San Francisco, located in the city of Quito, Ecuador.

Approval was formalized under code 031 on 16 August 2023. The study adhered to the ethical principle established in the Declaration of Helsinki and to current regulations in Ecuador.

All participants were informed about the objectives, procedures, and potential risks of the study. Participation was voluntary and formalized through the signing of an informed consent form. The confidentiality of personal data was guaranteed through anonymization and the use of identification codes for the management and analysis of the information collected.

## 3. Results

### 3.1. Characteristics of Study Participants

One hundred participants, 34 men and 66 women, completed the assessments. The average age was 49 years (women) and 50 years (men). Most were married, with a university degree and moderate monthly salaries. There was no gender variation in tobacco use (*p* = 0.06). However, women reported less physical activity than men (*p* = 0.01). See Table 1

CVR was mainly moderate in men and low in women. Men had higher blood pressure levels (130.7/81.6 mmHg vs. 121.9 /71.8 mmHg; *p* < 0.05) and higher values for weight (82.3 kg vs. 68.3 kg), waist circumference (103.5 cm vs. 92.9 cm) and muscle mass (31.3 kg vs. 22 kg); *p* < 0.05. In contrast, women had higher body fat percentage (41.1 vs. 31.9), and higher but non-significant values for total body fat (28.4 kg vs. 26.7 kg) and visceral fat (13.7 vs. 11.6). Waist-to-hip ratio and BMI were elevated in both sexes, with no significant differences.

In biochemical analysis, women had more hypertriglyceridemia (54.7%) and elevated LDL-c (66.7%), while in men, impaired fasting glucose (IFG) (47.1%) and elevated LDL-c (52.9%) were the most frequent. Other biochemical parameters remained within normal ranges in both groups.

### 3.2. Cardiovascular Risk Pre- and Post-Intervention

Table 2 shows the changes observed after the program, with increased physical activity and a reduced predicted risk CVR score in moderate-to-high-risk participants (≥5%) as well as key variables such as weight, BMI, WHR, body fat mass and visceral fat level, reflecting improvements in body composition. The metabolic profile showed clinically relevant changes by reducing glucose (*p* = 0.04) and increasing HDL (*p* = 0.022); however, there were no favorable changes in other lipids or smoking status. A summary of results as infographic is included in the Supplementary Material to facilitate a quick visualization of the most relevant results (Image S1).

In addition, the results on the size effect (Cohen’s d) revealed a moderate-low impact on cardiovascular risk (d = 0.410; 95% CI: 0.200 to 0.800). Regarding anthropometric indicators, weight showed a moderate-low effect (d = 0.42; 95% CI: 0.10 to 0.50), followed by BMI (d = 0.36; 95% CI: 0.16 to 0.56) and waist-to-hip ratio (d = 0.37; 95% CI: 0.16 to 0.57). Systolic blood pressure demonstrated a low but positive effect (d = 0.30; 95% CI: 0.10 to 0.50). Finally, the lipid profile and glucose exhibited a low or no effect.

Table 3 shows the significant diastolic blood pressure (DBP) increase: A statistically significant rise in DBP was observed in the overall analysis (−2.7 mmHg, *p* = 0.003). This change was addressed via a supplementary analysis by stratifying participants based on baseline DBP status (<90 mmHg vs. ≥90 mmHg). Pre–Post was used to compute the mathematical differences. The intervention resulted in a substantial clinical reduction of 6.083 mmHg (*p* = 0.049) in the subgroup with high baseline DBP (≥90 mmHg). Participants in the low/normal baseline subgroup (<90 mmHg), on the other hand, had an increase of 3.932 mmHg (*p* < 0.001). Rather than having a negative clinical impact, this increase is a physiological adjustment of readings that were previously below ideal levels, most likely indicating better cardiovascular efficiency after starting regular physical activity.

### 3.3. Cardiovascular Risk Pre and Post-Intervention in Men

When analyzing the data by sex (Table 4), the program showed significance after 4 months of intervention with notable decreases in weight (*p* = 0.003), abdominal circumference (*p* = 0.03) and visceral fat level (*p* = 0.02). Other variables also showed positive post-intervention effects such as waist-to-hip ratio (0.95 cm vs. 0.94 cm), body fat percentage (31.9% vs. 31.4%), body fat mass (26.7 vs. 25.6). The metabolic profile showed non-significant decreases in glucose and triglycerides, together with increases in LDL, HDL and total cholesterol. Non-Significant changes in blood pressure were also observed with decreased systolic (130.7 mmHg, vs.127.7 mmHg) and increased diastolic (81.6 mmHg vs. 82 mmHg) blood pressure. These changes are attributed to a subgroup of participants who showed abnormally low baseline TAD values, which normalized after the intervention; therefore, these variations are not of clinical significance.

The intervention showed moderate-low effects on key anthropometric variables. Weight showed a reduction with a d = 0.541 (95% CI: 0.177 to 0.898), and visceral fat was also pronounced (d = 0.412, 95% CI: 0.059 to 0.790). Other body composition indicators, such as abdominal circumference (d = 0.252) and fat mass (d = 0.366), also showed favorable changes of low to moderate magnitude.

### 3.4. Cardiovascular Risk Pre and Post-Intervention in Women

In women (Table 5) significant changes were observed in weight, BMI, waist-to-hip ratio and body fat mass (*p* < 0.05). Waist circumference (92.9 vs. 92), body fat percentage (41.1 vs. 40.4), visceral fat level (13.7 vs. 13.3) and LDL (107.9 mg/dL vs.107.8 mg/dL) showed favorable but non-significant decreases as did glucose (101.5 mg/dL vs. 99.5 mg/dL), and HDL (52.7 mg/dL vs. 54.9 mg/dL). Blood pressure also changed (121.9/72.8 mmHg vs. 117.8/ 76.8 mmHg), reflecting mixed but favorable changes in female CVR indicators.

Regarding the magnitude of the effect (Cohen’s d), the intervention showed a moderate-low impact on body composition variables. Specifically, reductions were observed in weight (d = 0.37; 95% CI: 0.12 to 0.62), body mass index (d = 0.41; 95% CI: 0.16 to 0.66), fat mass (d = 0.38; 95% CI: 0.13 to 0.63), and waist-to-hip ratio (d = 0.40; 95% CI: 0.15 to 0.65). As for clinical indicators, systolic blood pressure showed a slight decrease (d = 0.37; 95% CI: 0.12 to 0.62). Conversely, the impact on lipid profiles and glucose was low or negligible. with effect sizes that did not reach the thresholds of clinical relevance.

Participant attendance was moderate; 52% of the cohort had good attendance (**≥**6 sessions), whereas 48% of the cohort (n = 48) attended fewer than six sessions. An exploratory sensitivity analysis was performed to evaluate the potential dose-response effect (Table 6). Participants with good attendance had more pronounced changes in waist circumference (−2.7 cm vs. −1.6 cm), SBP (−3.9 mmHg vs. −3.4 mmHg), and body weight (−1.6 kg vs. −1.1 kg) than those in the low-attendance group. However, the Globorisk score dropped more in the low-attendance group (−0.8 vs. −0.06 points), which may be explained by the risk model’s non-linear structure, which is strongly impacted by categorical factors like age-specific coefficients and smoking status.

## 4. Discussion

The findings of this study suggest that the pragmatic implementation of, “Transforma tu vida con cambios diarios”, a workplace lifestyle modification program adapted from the gold-standard Diabetes Prevention Program (DPP), was associated with favorable reductions in multiple cardiovascular risk factors (CVRF) among Ecuadorian adults affiliated with the IESS. The intervention led to a statistically significant reduction in the mean cardiovascular risk score from 8.03 to 6.71 (*p* = 0.03) over 16 weeks, which corresponds to a moderate-low clinical impact (Cohen’s d: 0.410). This finding is crucial, as cardiovascular diseases (CVD) remain the leading cause of death globally and in Ecuador [1], and these preliminary results support the utility of workplace-based preventive strategies in underserved Latino populations

The overall results were associated with clinically meaningful improvements, including significant reductions in weight (from 73.1 to 71.7 kg, *p* < 0.001), BMI (from 29.0 to 28.5 kg/m^2^, *p*< 0.001) and body fat mass (from 27.8 to 26.8 kg, *p* < 0.001). While these changes were statistically robust, the associated effect size for weight (*d* = 0.424) and BMI (*d* = 0.363) indicate a moderate-low clinical magnitude. These anthropometric improvements align robustly with meta-analyses of behavioral interventions, which consistently report positive effects on body weight and composition [26]. Furthermore, the reduction in systolic blood pressure (SBP) (from 124.9 to 121.2 mmHg, *p* = 0.003) demonstrated a moderate-low clinical impact (*d* = 0.301), while metabolic markers like and in fasting glucose (from 103.3 to 101.1 mg/dL, *p* = 0.04) and HDL-cholesterol (from 51.5 to 54.9 mg/dL, *p* = 0.02) showed statistically significant but low effect size (*d* = 0.218 and *d* = −0.286, respectively)

While the observed reduction in the mean cardiovascular risk score from 8.03% to 6.71% (*p* = 0.03) is a statistically significant finding, its interpretation requires acknowledging the intrinsic methodological link to the intervention’s successful effects. Because the primary targets of the intervention, specifically the reduction in weight, BMI, and SBP, are direct inputs for the non-laboratory Globorisk model, the calculated 1.32 percentage point decline in the CVR score is primarily a mathematical consequence of achieving improvements in those specific metrics. This relationship, however, confirms the clinical value of the findings: the reduction in the CVR score serves as a powerful validation of the clinical significance of the observed anthropometric and hemodynamic improvements that the program’s lifestyle changes directly translate into a measurable reduction in calculated prognostic risk score, rather than providing evidence of an independent biological effect.

The observed association between the intervention and reduced risk factors may be linked to its strategic deployment within the workplace, which emerges as a critical setting for health promotion because most adults spend a substantial portion of their lives there The workplace provides an established structure that facilitates access to an at-risk yet organized population while maximizing adherence, as evidenced by the 100% retention rate achieved in this study. Delivering the intervention through companies affiliated with the IESS, allows for broad coverage of the formal working sector and mitigate recruitment barriers. Other studies suggest that workplace-based lifestyle interventions are associated with favorable CVR metrics, particularly when physical activity is integrated into the working day [27,28]. In the current study, the results regarding effect size revealed a moderate-low impact on anthropometric indicators, particularly for weight and BMI. Regarding clinical parameters, the intervention demonstrated a moderate-low effect on systolic blood pressure.

This program’s logistical adaptation, including the use of simple physical activity routines, adapting follow up visits to work schedules, and leveraging digital support via platforms like WhatsApp and Instagram for ongoing reinforcement, was crucial to overcoming barriers like lack of time or lack of perceived value, which often compromise adherence in corporate wellness initiatives. The reduction in physical inactivity prevalence (from 60% to 39%, *p* = 0.001) serves as notable indicator of the potential of this adapted model and provides preliminary evidence to inform the necessary transition from a reactive to a protective public health policy in Ecuador. Currently, Ecuadorian labor regulations, like the Regulation of Hygiene and Safety, are historically focused on preventing physical and occupational hazards, lacking an explicit and robust mandate for chronic non-communicable disease (NCD) prevention [29]. The observed improvements in CVR should serve as a basis for health authorities (Ministerium of Public Health, MSP) and the IESS to advocate for a legislative expansion of the Occupational Health mandate. This expansion should align with the regional strategies, such as the Health Promotion in the Workplace (HPW) initiative by PAHO, which emphasizes incorporating healthy lifestyle as strategic pillars [30]. Specifically, new legislation is needed to formally recognize and incentivize the involvement of health professionals (e.g., nutritionists, physical educators) in the delivery of these programs, securing their role within the social security system to ensure program sustainability and scale [31,32].

Sex-stratified analysis revealed distinct baseline profiles and response patterns that warrant careful interpretation. Men exhibited a predominantly moderate-to-high baseline CVR, characterized by significantly higher SBP (130.7 mmHg vs. 121.9 mmHg) and greater central adiposity (103.5 cm vs. 92.9 cm) compared to the lower-risk profile of women. Notably, men achieved a significant reduction in visceral fat (*d* = 0.412, *p* = 0.02), a finding consistent with sex dimorphism in adipose tissue function. While women’s subcutaneous fat often offers relative metabolic protection, the focal reduction in visceral fat among men targets the most metabolically deleterious component, providing compelling evidence for the utility of sex-specific monitoring in future workplace initiatives [33,34,35].

While cultural adaptation typically integrates ethnic values and cosmovisions, this program utilized contextual, logistical, and linguistic tailoring to ensure feasibility within the Ecuadorian workplace. [36]. By adjusting schedules, delivery formats, and nutritional advice to local habits, the program achieved a moderate overall clinical effect in CVR score (*d* = 0.410), suggesting that contextual adaptation is operationally effective for this formal labor sector cohort. To maintain scientific rigor, the intervention is best described as an “adaptation to the Ecuadorian context and operational environment”. However, for the program to be equitable across Ecuador’s diverse regions, future research must address interculturality. For instance, the Andean cosmovision links health to energetic and social balances, necessitating a shift toward narrative-based medicine to reach populations beyond westernized labor sector [37]. To truly reach all populations, especially those underserved, future DPP adaptations must move towards medicine based on narratives, integrating local worldviews, as advocated for health promotion in diverse cultural settings [38,39].

However, a meta-analysis indicates that Hispanic-focused treatments, including cuisine, culture, family, and community, enhance weight loss (effect size 0.01 to 0.32). Workplace programs with medical guidance, regular contact, and motivational support also significantly improve CVR markers [8,40]. Longer durations may increase metabolic benefits, but 12- to 16-week interventions yield better weight outcomes than shorter ones [10,41]. Workplace delivery provides strategic advantages, especially for marginalized groups, given long working lifespans [8].

On the other hand, smoking prevalence dropped from 12% to 9%. Though not statistically significant, this clinically relevant reduction may lower CVR, with quitting reducing recurrent events by one-third, highlighting its importance for cardiovascular health [42]. Culturally tailored cessation techniques, supported by social and environmental factors, enhance lifestyle program efficacy in high-risk Latino populations [43].

Regarding the moderate adherence rate (52%), it highlights the practical challenges of implementing lifestyle modifications within the formal labor sector. However, the exploratory sensitivity analysis confirmed the internal validity of the intervention because objective anthropometric and hemodynamic markers displayed a clear dose-response signal. The larger degree of physical improvements among participants who finished six sessions or more suggests that direct exposure to the program’s workshops and follow-up visits is a significant determinant in success. The Globorisk score showed a greater decline in the low-attendance group, while clinical markers exhibited a clear dose-response trend, with higher attendance leading to greater decreases in weight and blood pressure, according to the exploratory sensitivity analysis. The CVR model, which is highly weighted by variables like smoking status and age-specific coefficients, is probably the cause of this disparity. Because the continuous physiological markers in the high-attendance cohort more accurately reflected the overall effectiveness of the lifestyle modifications, the calculated risk reduction in the low-attendance subgroup may represent individual differences in high-impact risk factors.

### Limitations and the Efficacy of Components

A key limitation of this quasi-experimental, pre-post design is the inability to isolate the independent effects of individual intervention components, as physical activity and dietary modifications were delivered simultaneously within a multifactorial lifestyle program. Nevertheless, the pattern of observed outcomes provides relevant insight into the relative contribution of each component. The most pronounced effects were observed in variables closely associated with increased physical activity. The significant reduction in physical inactivity was accompanied by meaningful improvement in systolic blood pressure, which is consistent with the well-established CV benefits of regular exercise. In contrast, reductions in fasting glucose were statistically significant but modest in magnitude, suggesting a comparatively lower impact of the dietary component, potentially related to the short intervention duration and challenges in sustaining changes outside controlled settings [44,45,46].

Another limitation of our quasi-experimental study is the lack of a control group which limits causal inference; however, the consistent direction of changes across multiple CV risk markers supports the internal validity of the findings. Importantly, the stratified analysis of diastolic blood pressure clarified that the overall increase observed in aggregated analyses reflected physiological normalization among participants with low baseline values, whereas individuals with elevated baseline DBP experienced a clinically beneficial reduction. Nonetheless, well-designed pre-post studies can indicate causal effects in populations with known baseline risks. The significant improvements support the short-term efficacy of the intervention; however, the 16-week follow-up limits conclusions regarding its long-term sustainability. Meta-analyses indicate lifestyle interventions effects on biological markers may decline over time, while behavioral changes tend to persist, highlighting the importance of distinguishing short-term efficacy from long-term impact in evaluating lifestyle programs [47]. Future research should use longer follow-up periods to assess the sustainability of CVR reductions. Additionally, the absence of a formal pre- and post-intervention knowledge assessment is a constraint, and attendance was used as a pragmatic proxy of exposure to the intervention rather than as a measure of educational results. Therefore, objectively measured clinical, anthropometric, and behavioral pre-post changes were the main means of evaluating effectiveness.

Finally, the relatively short follow-up period and modest sample size limit conclusions regarding long-term sustainability and the detection of smaller metabolic effects. Despite these constraints, results contribute to the growing body of evidence supporting tailored approaches in high-risk groups, showing improvements in body weight, blood pressure, HDL cholesterol, and overall cardiovascular risk, supporting the effectiveness of pragmatic, culturally adapted workplace interventions in reducing CV risk in high-risk Latino populations [48].

## 5. Conclusions

This 16-week, culturally adapted, workplace-based lifestyle intervention greatly improved key cardiometabolic markers in a high-risk Latino working group. Body weight (−1.4 kg), body mass index (−0.5 kg/m^2^), systolic blood pressure (−3.7 mmHg), HDL cholesterol (+3.4 mg/dL), fasting glucose (−2.2 mg/dL), 10-year cardiovascular risk (reduction of 1.3 percentage points), and the prevalence of physical inactivity (reduction from 60% to 39%). According to stratified analyses, individuals with high baseline diastolic blood pressure had clinically significant drops, whereas those with low baseline values showed increases that were more suggestive of physiological normalization than detrimental effects. These findings all suggest that realistic, context-adapted workplace interventions could be useful as scalable preventive measures in high-risk Latin American environments. Modest anthropometric, hemodynamic, and behavioral improvements can be transformed into measurable decreases in predicted cardiovascular risk scores by these interventions.

## Figures and Tables

**Table 1:** Baseline sociodemographic, clinical, behavioral, anthropometric, and biochemical data in participants, differentiated by sex (n = 100).

Variables		Male	Female	*p-*Value
		(n = 34)	(n = 66)	
Age, years, m (SD)		50 (6)	49 (6)	0.52
Education, n (%)	Lower than high school	2 (5.9)	0 (0)	0.11
	High school	7 (20.6)	8 (12.1)	
	College	17 (50)	34 (51.5%)	
	Graduate	8 (23.5)	24 (36.4)	
Marital status, n (%)	Single	2 (5.9)	16 (24.2)	0.002
	Married	28 (82.4)	33 (50)	
	Divorced	1 (2.9)	12 (18.2)	
	Widowed	0 (0)	4 (6.1)	
	De facto union	3 (8.8)	1 (1.5)	
Monthly income level, n (%)	1 to 2 basic salaries	22 (64.7)	49 (74.2)	0.51
	3 to 4 basic salaries	9 (26.5)	11 (16.7)	
	5 or more basic salaries	3 (8.8)	6 (9.1)	
Current smoking, n (%)	No	27 (79.4)	61 (92.4)	0.06
	Yes	7 (20.6)	5 (7.6)	
Weekly physical activity Level, n (%)	Low	14 (41.2)	46 (69.7)	0.01
	Moderate	7 (20.6)	16 (24.2)	
	High	13 (38.2)	4 (6.1)	
Cardiovascular risk level, n (%)	Low	10 (29.4)	55 (83.3)	<0.001
	Moderate	14 (41.2)	10 (15.2)	
	High	10 (29.4)	1 (1.5)	
	Very high	0 (0)	0 (0)	
Weight, kg, m (SD)		82.3 (10.4)	68.3 (10.5)	<0.001
SBP, mmHg, m (SD)		130.7 (16.1)	121.9 (14.7)	0.01
DBP, mmHg, m (SD)		81.6 (10)	72.8 (11)	<0.001
BMI, kg/m^2^, m (SD)		29.3 (3.2)	28.8 (3.7)	0.44
Waist circumference, cm, m (SD)		103.5 (14.4)	92.9 (8.5)	<0.001
WHR, m (SD)		0.95 (0.04)	0.94 (0.04)	0.39
Muscle mass, kg, m (SD)		31.3 (4.6)	22.0 (3.4)	<0.001
Body fat percentage, %, m (SD)		31.9 (5.6)	41.1 (5.0)	<0.001
Body fat mass, kg, m (SD)		26.7 (6.3)	28.4 (7.0)	0.23
Visceral fat level, m (SD)		11.6 (3.6)	13.7 (3.5)	0.004
Glucose, mg/dL, m (SD)		106.6 (20.6)	101.5 (14.2)	0.19
Glucose ranges, n (%)	Normal < 100	14 (41.2)	37 (56.1)	0.16
	PDR 100–125	16 (47.1)	27 (40.9)	
	DR ≥ 126	4 (11.8)	2 (3.0)	
Total cholesterol, mg/dL, m (SD)		193.9 (51.9)	187.7 (35.8)	0.49
Cholesterol ranges, n (%)	Normal < 200	18 (52.9)	39 (59.1)	0.56
	High ≥ 200	16 (47.1)	27 (40.9)	
Triglycerides, mg/dL, m (SD)		182.7 (96.6)	170.1 (74.4)	0.91
Triglycerides ranges, n (%)	Normal < 150	17 (53.1)	29 (45.3)	0.47
	High ≥ 150	15 (46.9)	35 (54.7)	
HDL-c, mg/dL, m (SD)		49.2 (10.9)	52.7 (11.0)	0.33
Female HDL-c, n (%)	Normal ≥ 50	-	38 (57.6)	
	Low < 50		28 (42.4)	
Male HDL-c, n (%)	Normal ≥ 40	0 (0)	-	
	Low < 40	28 (100)		
LDL-c, mg/dL, m (SD)		102.8 (38.3)	107.6 (27.8)	0.49
LDL-c ranges, n (%)	Normal < 100	16 (47.1)	22 (33.3)	0.18
	High ≥ 100	18 (52.9)	44 (66.7)	

m: mean; SD: standard deviation; PDR: prediabetic range; DR: diabetic range; SBP: systolic blood pressure; DBP: diastolic blood pressure; BMI: body mass index; WHR: waist-to-hip ratio; HDL-c: high density lipoprotein cholesterol; LDL-c: low density lipoprotein cholesterol.

**Table 2:** Behavioral, clinical, anthropometric, and biochemical data pre intervention and postintervention in study participants, overall (n = 100).

Variables	Pre	Post	*p*-Value	Cohen’s d	95% IC	
	Intervention	Intervention				
					Lower	Upper
Smoke, n (%)						
No	88 (88)	91 (91)	0.25			
Yes	12 (12)	9 (9)				
Weekly physical activity level, n (%)						
Low	60 (60)	39 (39)	0.001			
Moderate/High	40 (40)	61 (61)				
Cardiovascular risk, m (SD)	8.03 (3.2)	6.71 (3.2)	0.03	0.410	0.200	0.800
Weight, kg, m (SD)	73.1 (12.3)	71.7 (12.2)	<0.001	0.424	0.219	0.628
BMI, kg/m^2^, m (SD)	29 (3.5)	28.5 (3.7)	<0.001	0.363	0.160	0.564
Waist circumference, cm, m (SD)	96.5 (11.9)	94.3 (8.9)	0.01	0.252	0.052	0.450
SBP, mmHg, m (SD)	124.9 (15.7)	121.2 (14.9)	0.003	0.301	0.100	0.501
DBP, mmHg, m (SD)	75.8 (11.4)	78.5 (10)	0.003	−0.303	−0.503	−0.102
WHR, m (SD)	0.94 (0.04)	0.93 (0.04)	<0.001	0.367	0.164	0.569
Muscle mass, kg, m (SD)	25.2 (5.8)	24.8 (5.7)	0.20	0.131	−0.067	0.327
Body fat percentage, %, m (SD)	37.9 (6.8)	37.4 (7)	0.05	0.198	0.000	0.395
Body fat mass, kg, m (SD)	27.8 (6.81)	26.8 (6.84)	<0.001	0.366	0.163	0.568
Visceral fat level, m (SD)	13 (3.7)	12.5 (4)	0.01	0.262	0.062	0.461
Glucose, mg/dL, m (SD)	103.3 (16.7)	101.1 (15.8)	0.04	0.218	0.019	0.416
Total cholesterol, mg/dL, m (SD)	189.7 (41.7)	196.6 (35.6)	0.05	−0.196	−0.395	0.090
Triglycerides, mg/dL, m (SD)	174.3 (82.2)	180.8 (88.8)	0.28	−0.111	−0.311	0.495
HDL-c, mg/dL, m (SD)	51.5 (11.1)	54.9 (10.1)	0.02	−0.286	−0.488	−0.082
LDL-c, mg/dL, m (SD)	106.5 (31.9)	106.7 (28.7)	0.97	−0.004	−0.211	0.202

m: mean; SD: standard deviation; PDR: prediabetic range; DR: diabetic range; SBP: systolic blood pressure; DBP: diastolic blood pressure; BMI: body mass index; WHR: waist-to-hip ratio; HDL-c: high density lipoprotein cholesterol; LDL-c: low density lipoprotein cholesterol.

**Table 3:** Variations in diastolic blood pressure.

Baseline DBP Status	Range	Mean Change (Pre-Post)	*p*-Value	Clinical Interpretation
Low/Normal DBP	DBP < 90 mmHg	−3.932 mmHg ↑	<0.001	Significant rise (normalization)
High DBP	DBP ≥ 90 mmHg	+6.083 mmHg ↓	0.049	Significant reduction (positive clinical benefit)

**Table 4:** Clinical, anthropometric and biochemical data pre intervention and postintervention in male participants in the study. (n = 34).

Variables	Pre	Post	*p*-Value	Cohen’s d	95% CI	
	Intervention	Intervention				
					Lower	Upper
Weight, kg, m (SD)	82.3 (10.4)	80.8 (10.7)	0.003	0.541	0.177	0.898
BIM, kg/m^2^, m (SD)	29.3 (3.2)	29 (3.4)	0.14	0.257	−0.087	0.597
Waist circunference, cm, m (SD)	103.5 (14.4)	98.8 (9.4)	0.03	0.390	0.039	0.737
SBP, mmHg, m (SD)	130.7 (16.1)	127.7 (12.6)	0.24	0.207	−0.134	0.545
DBP, mmHg, m (SD)	81.6 (10.4)	82 (9.2)	0.81	−0.041	−0.377	0.296
WHR, m (SD)	0.95 (0.04)	0.94 (0.04)	0.10	0.286	−0.059	0.627
Muscle mass, kg, m (SD)	31.25 (4.6)	31.26 (4.3)	0.97	−0.006	−0.342	0.320
Body fat percent, %, m (SD)	31.9 (5.6)	31.4 (6.1)	0.30	0.181	−0.159	0.519
Body fat mass, kg, m (SD)	26.7 (6.3)	25.6 (6.6)	0.05	0.344	−0.005	0.688
Visceral fat level, m (SD)	11.6 (3.6)	11 (3.6)	0.02	0.412	0.059	0.760
Glucose, mg/dL, m (SD)	106.6 (20.6)	104.3 (16.1)	0.21	0.213	−0.129	0.551
Total cholesterol, mg/dL, m (SD)	193.8 (51.9)	196.2 (45)	0.68	−0.073	−0.404	0.263
Triglycerides, mg/dL, m (SD)	182.7 (96.6)	171.8 (82.5)	0.49	0.147	−0.202	0.495
HDL-c, mg/dL, m (SD)	49.6 (10.9)	54.9 (10.5)	0.06	−0.415	−0.773	−0.050
LDL-c, mg/dL, m (SD)	103.8 (38.6)	104.3 (36.3)	0.93	−0.017	−0.374	0.341

m: mean; SD: standard deviation; PDR: prediabetic range; DR: diabetic range; SBP: systolic blood pressure; DBP: diastolic blood pressure; BMI: body mass index; WHR: waist-to-hip ratio; HDL-c: high density lipoprotein cholesterol; LDL-c: low density lipoprotein cholesterol.

**Table 5:** Clinical, anthropometric and biochemical data pre intervention and postintervention in female participants in the study. (n = 66).

Variables	Pre Intervention	Post Intervention	*p*–Value	Cohen’s d	95% CI	
					Lower	Upper
Weight, kg, m (SD)	68.3 (10.5)	67 (10.2)	0.003	0.374	0.123	0.622
BMI, kg/m^2^, m (SD)	28.8 (3.7)	28.2 (3.8)	0.001	0.415	0.162	0.665
Waist circumference, cm, m (SD)	92.9 (8.5)	92 (7.9)	0.24	0.147	−0.096	0.389
SBP, mmHg, m (SD)	121.9 (14.7)	117.8 (15)	0.004	0.369	0.118	0.617
DBP, mmHg, m (SD)	72.8 (10.7)	76.8 (10)	<0.001	−0.478	−0.731	−0.221
WHR, m (SD)	0.94 (0.04)	0.93 (0.05)	0.002	0.405	0.152	0.655
Muscle mass, kg, m (SD)	22 (3.4)	21.5 (2.8)	0.12	0.195	−0.050	0.437
Body fat percentage, %, m (SD)	41.1 (5)	40.4 (5.2)	0.10	0.205	−0.040	0.448
Body fat mass, kg, m (SD)	28.4 (7)	27.4 (6.9)	0.003	0.378	0.127	0.627
Visceral fat level, m (SD)	13.7 (3.5)	13.3 (3.9)	0.08	0.218	−0.027	0.461
Glucose, mg/dL, m (SD)	101.5 (14.2)	99.5 (15.5)	0.09	0.220	−0.025	0.463
Total cholesterol, mg/dL, m (SD)	187.7 (35.8)	196.7 (30.2)	0.05	−0.252	−0.496	−0.006
Triglycerides, mg/dL, m (SD)	170.1 (74.4)	185.4 (92.1)	0.08	−0.194	−0.441	0.059
HDL-c, mg/dL, m (SD)	52.7 (11)	54.9 (9.98)	0.14	−0.218	−0.463	0.029
LDL-c, mg/dL, m (SD)	107.9 (28.3)	107.8 (24.4)	0.99	0.002	−0.251	0.255

m: mean; SD: standard deviation; PDR: prediabetic range; DR: diabetic range; SBP: systolic blood pressure; DBP: diastolic blood pressure; BMI: body mass index; WHR: waist-to-hip ratio; HDL-c: high density lipoprotein cholesterol; LDL-c: low density lipoprotein cholesterol.

**Table 6:** Comparison Comparing clinical outcomes between adherence subgroups (6 vs. <6 sessions) using an exploratory sensitivity analysis.

Outcome Variable	Mean Change in ≥6 Sessions Group	Mean Change in <6 Sessions Group	Dose-Response Trend
Body Weight (kg)	−1.6	−1.1	Positive
SBP (mmHg)	−3.9	−3.4	Positive
WC (cm)	−2.7	−1.6	Positive
Globorisk Score	−0.06 points	−0.8 points	Inconsistent (Model sensitivity)

SBP: systolic blood pressure; WC: waist circumference.

## Data Availability

The datasets generated and/or analysed during the present study are not publicly available because the data are not public. However, they may be requested from the corresponding author upon reasonable request.

## Supplementary Materials

The following supporting information can be downloaded at: https://www.mdpi.com/article/10.3390/jcm15020628/s1, Table S1: Description of the sessions; Booklet S1: Standardized booklet.; Image S1: Infographic of the pertinent result findings.

## Abbreviations

The following abbreviations are used in this manuscript:

CVD	Cardiovascular diseases
CVR	Cardiovascular risk
T2D	Type 2 diabetes mellitus
INEC	Instituto Nacional de Estadísticas y Censos
DPP	Diabetes Prevention Program
CDC	Centers for Disease Control and Prevention
IESS	Instituto Ecuatoriano de Seguridad Social
MSP	Ministerio de Salud Pública
IPAQ	International Physical Activity Questionnaire
M	Mean
SD	Standar deviation
PDR	Prediabetic range
DR	Diabetic range
SBP	Systolic blood pressure
DBP	Diastolic blood pressure
BMI	Body mass index
WHR	Waist-to-hip ratio
HDL-c	High density lipoprotein cholesterol
LDL-c	Low density lipoprotein cholesterol
