# Supplementary material for: Impact of a Contextualized Workplace Intervention in a Latino Population on Reducing Cardiovascular Risk and Its Associated Factors
Source: J Clin Med. 2026 Jan 13;15(2):628. doi: 10.3390/jcm15020628 (PMC12841714; doi:10.3390/jcm15020628)

PROGRAMME STRUCTURE "Trasnforma tu vida con cambios diarios"

1. General structure and Dose intervention
The intervention was a quasi-experimental pre-post study conducted over 14 weeks in the participants' workplaces. The program followed a hybrid model of face-to-face and digital delivery to ensure adherence:

- ✓ **In-person Workshops:** Two major workshops (45-minute rotating stations) led by physicians and registered dietitians.
- ✓ **Follow-up Visits:** Four on-site visits (Sessions 4, 8, 9, and 10) during working hours, coordinated with Human Resources.
- ✓ **Digital Support:** Continuous accompaniment via WhatsApp and Instagram to provide educational videos, key slides from the face-to-face session topics and motivational messages based on the adapted session topics and session reminders.

2. Details of the Sessions

<u>Pillar A:</u> Nutritional Education and Metabolic Awareness

The program focused on specific clinical literacy and practical tools:

- ✓ **Clinical Correlation:** Participants reflected on morbidity and mortality statistics in Ecuador to understand the family and social impact of cardio-metabolic diseases.
- ✓ **Fat Quality Identification:** Practical exercises using household measures and scales to estimate fat and calorie content in common foods.
- ✓ **Label Reading:** Training on the "nutritional traffic light" to identify high-risk processed foods.
- ✓ **Caloric Balance:** A "Tipping the Scales" game where participants classified daily actions that increase or decrease energy balance.
- ✓ Mindful Eating: Guided simulations to practice eating slowly and recognizing physiological satiety signals.

<u>Pillar B:</u> Physical Activity Prescription

The intervention transitioned from sedentary behavior to a structured plan:

- ✓ FITT Framework: Participants were taught to calculate Frequency, Intensity, Type, and Time of exercise.
- ✓ Intensity Self-Monitoring: Use of the "Talk Test" and manual heart rate measurement to identify the target training zone (50–70% of maximum heart rate HR max).
- ✓ Goal Setting: A commitment to reach 150 minutes of weekly activity, starting from a baseline of 60 minutes, recorded in a standardized printed booklet.
- ✓ Spontaneous Movement: Creation of a "Daily Active Actions" list (e.g., using stairs instead of elevators, walking to work).
- ✓ Safety Protocols: Education on warning signs (chest pain, dyspnea, dizziness) requiring immediate cessation of exercise.

<u>Pillar C:</u> Behavioral and Cognitive Strategies

To ensure adherence to the program included psychological tools:

- ✓ Stimulus Control: Identification of "problem signals" (environmental cues) in the workplace and home that trigger poor eating or sedentary habits.
- ✓ Cognitive Restructuring: Exercises to identify self-sabotaging thoughts (e.g., "I don't have time") and replace them with positive affirmations.
- ✓ Social Engineering: Role-playing exercises to rehearse healthy responses to social pressure during parties or family gatherings.
- ✓ Stress Management: Training in deep breathing techniques and "active breaks" as coping mechanisms for workplace stress.

3. Implementation Tools for the Workplace

The following materials were central to the intervention's:

- ✓ Standardized Booklet: A printed logbook where participants registered weight, physical activity type/duration, daily step counts, and personal goals.
- ✓ Visual Models: Use of food models during workplace visits to facilitate nutritional counseling.
- ✓ Digital Alternatives: For participants unable to attend in person due to work schedules, all information was delivered through digital platforms to maintain dose consistency.

Supplementary Note: Digital Strategy and Remote Adaptation

To ensure replicability and maintain the intervention dose in a dynamic work environment, a complementary digital strategy was implemented:

- ✓ **Continuous Accompaniment:** A WhatsApp group and an Instagram account were created for informational purposes to support participants in their process of change.
- ✓ **Multimedia Content**: The research team developed and provided educational material adapted from the session topics, including videos and slides.
- ✓ **Logistics Management:** These platforms were used to plan and coordinate follow-up visits to workplaces, optimising time during the working day.
- ✓ **Asynchronous Intervention**: Participants who were unable to attend the face-to-face sessions due to work schedules received all the information through these digital media to ensure equal access to the programmer.
- ✓ **Digital Reinforcement**: Digital support served to reinforce the achievements recorded in the standardised printed brochure provided at the start of the programmer.

Table S1. Description of the sessions

	**Sessions**	**Topics covered**	**Activities carried out**
Workshop 1	**SESSION 1: Welcome**	Cardiometabolic disease. Reflection on the importance of making lifestyle changes.	**Presentation:** Statistics on cardio-metabolic morbidity and mortality in Ecuador. Participants' reflections on cardio-metabolic diseases and how they affect the family and social environment. **Goal setting:** Participants were given a booklet to record their goals.
	**SESSION 2: Good food, good**	Recognition of healthy and unhealthy fats.	**Comparative food analysis:** participants identified foods

	**life**	Importance of macronutrient balance.<br>Identification of good quality food sources.	high and low in fat and calories using tables and practical examples.<br>**Observation exercise:** nutritional labels (nutritional traffic lights) were reviewed to recognise types of fat and food quality.<br>**Guided discussion:** participants reflected on how to replace low-quality foods with healthier options.<br>**Practical exercise:** participants estimated and measured the fat and calorie content of different foods using household measures and scales.
	**SESSION 3: Activate your body, activate your mind**	Motivation to start and maintain an active lifestyle.<br>Development of safe, progressive and enjoyable physical routines aimed at preventing cardio-metabolic diseases.	**Initial check-up:** their body weight was recorded to monitor their progress.<br>**Discussion on keys to success and safety:** The benefits of exercise on body composition, bone health, blood pressure, blood sugar, and lipid profile were discussed, and guidance was provided on the principles of safely starting physical activity regardless of current fitness level. In addition, the warning signs that require stopping exercise (chest pain, shortness of breath, dizziness, etc.) were explained.<br>**Personalized weekly activity planning:** Participants designed their own physical activity schedule, distributing 150 minutes per week into short sessions.<br>**Personalized weekly activity planning:** Participants designed their own physical activity schedule, distributing 150 minutes per week into short sessions.<br>The emphasis was on enjoyable and sustainable activities (walking, dancing,

			cycling).<br>**Monitoring and self-assessment:**<br>Training in the use of step-counting apps (Google Fit) and manual recording.<br>Calculation of daily average steps and comparison between working days and weekends.<br>Self-diagnosis dynamics<br>Individual questionnaire on current level of physical activity, personal barriers and motivators for change.<br>Practice in calculating intensity (FITT principle)<br>Guided exercise to calculate the frequency, intensity, type and time (FITT) of physical activity.<br>Practical measurement of heart rate and recognition of the target training zone (50–70% HR max).<br>**Practical activity:** "Measure your effort"<br>Participants measured their pulse at rest and during a short walk.<br>They applied the "talk test" to identify moderate intensity.<br>Spontaneous movement workshop<br>Participatory list of active daily activities (using stairs, walking to work, gardening, etc.).<br>Group commitment to incorporate at least three of these activities into the daily routine.<br>**Guided warm-up and stretching practice:**<br>Demonstration of joint mobility, breathing and stretching exercises.<br>Viewing videos and supervised performance of basic stretches (neck, back, limbs).

			Homework assignment<br>Commitment to perform at least 60 minutes of physical activity per week, gradually increasing to 150 minutes.<br>Recording in a booklet of the type, duration and days of exercise, along with the number of steps and heart rate.
Visit	**SESSION 4: Discover your inner motivation.**	Recognition and modification of environmental cues that influence eating habits and physical activity.<br>Development of personal strategies to maintain healthy behaviours.	**Individual progress monitoring:**<br>Recording body weight and updating personal charts.<br>Reviewing previous goals and providing feedback on achievements and difficulties.<br>**Guided reflection on habits and environment:**<br>Identifying "problem signals" related to food and sedentary behaviour at home, work, and shopping locations.<br>Group discussion on strategies to modify them (e.g., changing routes, avoiding impulse purchases, placing visual reminders).<br>**Designing new positive cues:** Participants created visual or environmental reminders that promote physical activity (notes on the television, alarms, exercise channel subscriptions).<br>**Practical problem-solving exercise (5 steps):**<br>Application of a structured methodology to identify obstacles, analyse options, choose solutions, create an action plan, and evaluate results.<br>**Group case study:** analysis of the "chain of action" of a fictional participant (Sara) to recognize links of risk.<br>**Workshop on managing thoughts and emotions:**<br>Analysis of "slips" or relapses in behavioural changes.

			Exercise to identify negative thoughts and replace them with positive affirmations using shared examples.<br>**Group activity:** each participant wrote down frequent self-critical thoughts and the group suggested positive or realistic responses.<br>**Homework:**<br>Replace a problematic food cue with a positive one.<br>Record progress and personal action plan in the programmer booklet.
Workshop 2	**SESSION 5:**<br>**Balance your calories, balance your life**	Energy balance: calories consumed vs. calories expended.<br>Identification of foods with high calorie density.<br>Conscious choice and moderation in portion sizes.	**Demonstration with examples:** comparison of products on the market with the same number of calories but different nutritional qualities (e.g. mayonnaise vs. avocado, soft drinks vs. water).<br>**Practical calculation:** participants learned to estimate their daily calorie balance using examples of meals and physical activities.<br>"Tipping the scales" game: group activity in which participants classify actions that increase or reduce energy balance.<br>**Individual reflection:** identification of possible personal changes to balance calories in their routine.<br>**Planning exercise:** creating a balanced sample menu based on the healthy plate model.
	**SESSION 6:**<br>**Be stronger than your excuses. Exercises to maintain an active lifestyle**	Progression strategies, self-control and enjoyment to consolidate regular physical activity habits.<br>Safety and injury prevention.	**Review of weekly goals:**<br>Reminder of the 150-minute/week goal and review of personal progress with examples of progression (from 60 to 150 minutes/week in 4 weeks).<br>**Development of a personal exercise plan:**<br>Each participant completed a weekly planning table,

			specifying activity, frequency, and daily duration.<br>**Practical demonstration of types of exercise:**<br>Identification of moderate aerobic activities (walking, dancing, cycling, swimming, gardening, hiking).<br>Discussion on the importance of strength training as a metabolic supplement.<br>**Awareness exercise:** "Move without excuses"<br>Group dynamic where participants shared common barriers and formulated practical solutions to maintain consistency.<br>**Practical warm-up and stretching session:**<br>Supervised execution of a complete routine of 11 stretches, including arms, torso, back, quadriceps and Achilles tendon.<br>Principles of controlled breathing, correct posture and personal limits without pain were applied.<br>Safety training and injury prevention<br>Review of the correct use of sports footwear.<br>Instruction on hydration, gradual progression and recognition of warning signs during exercise.<br>**Educational exercise:** "Listen to your body"<br>Simulation of warning signs (chest pain, fatigue, dizziness) and discussion about the appropriate response to each one.<br>**Motivational closing:** Group reflection on the psychological and metabolic benefits of exercise.<br>**Key message:** "No pain, no gain" – prioritise safety and consistency over intensity.

	**SESSION 7: Consistency is key: how to stay the course**	Adherence to behavioural changes.<br>Social and emotional strategies that facilitate consistency and reduce the risk of relapse.	**Analysis of social cues:**<br>Identification of problematic social cues (social pressure to eat, sedentary environments, negative comments) and helpful social cues (positive role models, support from family or friends).<br>Individual recording of personal examples.<br>**Changing social cues:**<br>Group discussion on four strategies for managing social influences:<br>Avoid tempting cues.<br>Modify the environment or negotiate changes.<br>Respond assertively and healthily.<br>Add positive social cues (such as walking buddies, shared healthy dinners).<br>**Planning exercise:**<br>Each participant developed an action plan to replace a negative social cue with a positive one.<br>**Simulation of social situations:**<br>Role-play events such as parties, holidays or family gatherings to rehearse healthy responses to food temptations or inactivity.<br>**Reflection on motivation:**<br>Review of the benefits achieved so far.<br>Recording personal achievements and drawing up a list of goals for the next six months.<br>**Stress management:**<br>Identification of personal sources of stress and discussion of strategies to control it (saying "no", delegating tasks, setting realistic goals, taking active breaks).<br>Brief relaxation and mindful breathing exercise.

			**Behavioural commitment:** Setting new physical activity goals (≥150 min/week) and weekly weight self-monitoring.
Visit	**SESSION 8: Savour every bite: tips for mindful eating and portion control**	Mindful eating.<br>Techniques for eating slowly and recognising signs of fullness.<br>Strategies for controlling portions and avoiding overeating.<br>Healthy choices when eating out.	**Guided simulation:** practising eating slowly, savouring food and recognising when you are full.<br>**Portion control exercise:** using the palm of your hand, measuring cups and a healthy plate to estimate appropriate portions.<br>**Menu analysis:** comparison between home-cooked meals and restaurant meals in terms of calorie content and fat quality.<br>**Group activity "Eating out":** discussion of strategies for ordering healthy options in restaurants.<br>**Self-assessment:** recording mindful eating habits and personal suggestions for improvement.
	**SESSION 9: Maintain balance: tips for a healthy and sustainable lifestyle in the long term**	Self-control and emotional management skills to maintain healthy habits and prevent stress and long-term relapses.	**Identifying counterproductive thoughts:** Group dynamics to recognise phrases or beliefs that sabotage weight control ("I don't have time", "I've already ruined my progress").<br>**Cognitive restructuring:** Exercise to replace negative thoughts with positive or self-affirming responses.<br>Individual recording of personal reinforcement phrases.<br>**Reflection on achievements:** Recognition of individual progress and giving personal credit for sustained effort.<br>**Stress management strategies:** Discussion of everyday sources of tension and application of techniques to

			prevent stress (deep breathing, active breaks, time management, social contact).<br>**Promotion of overall balance:**<br>Discussion on the importance of adequate sleep, relaxation, laughter, and enjoying the process of change as part of overall well-being.
	**SESSION 10: Don't give up: how to persevere when motivation starts to wane**	Perseverance in the face of loss of motivation.<br>Encouragement of self-reflection.<br>Sustainable personal commitments.	**Assessment of current physical activity:**<br>Participants classified their daily activities according to intensity (high or low) and compared active time with hours of sedentary behaviour per week.<br>**Analysis of inactivity and alternatives:**<br>Identification of sedentary moments and design of short substitute activities to reduce sitting time.<br>**Personal reflection and motivational closure:**<br>Writing a "personal story of healthy change," in which each participant highlighted the lessons learned and strategies that helped them overcome moments of discouragement.<br>Crafting a motivational message for themselves as a reminder of their achievements and commitment to their future well-being.

Booklet S1. Standardized booklet.

UTPL
La Universidad Católica de Loja

GRUPO INVESTIGACIÓN PREVENCIÓN
Y PROMOCIÓN EN SALUD
INVESTIGACIÓN PARA VIDAS SALUDABLES

Un pequeño cambio positivo puede cambiar tu día o tu vida entera. (Nishant Grover)

Transforma tu vida con cambios diarios

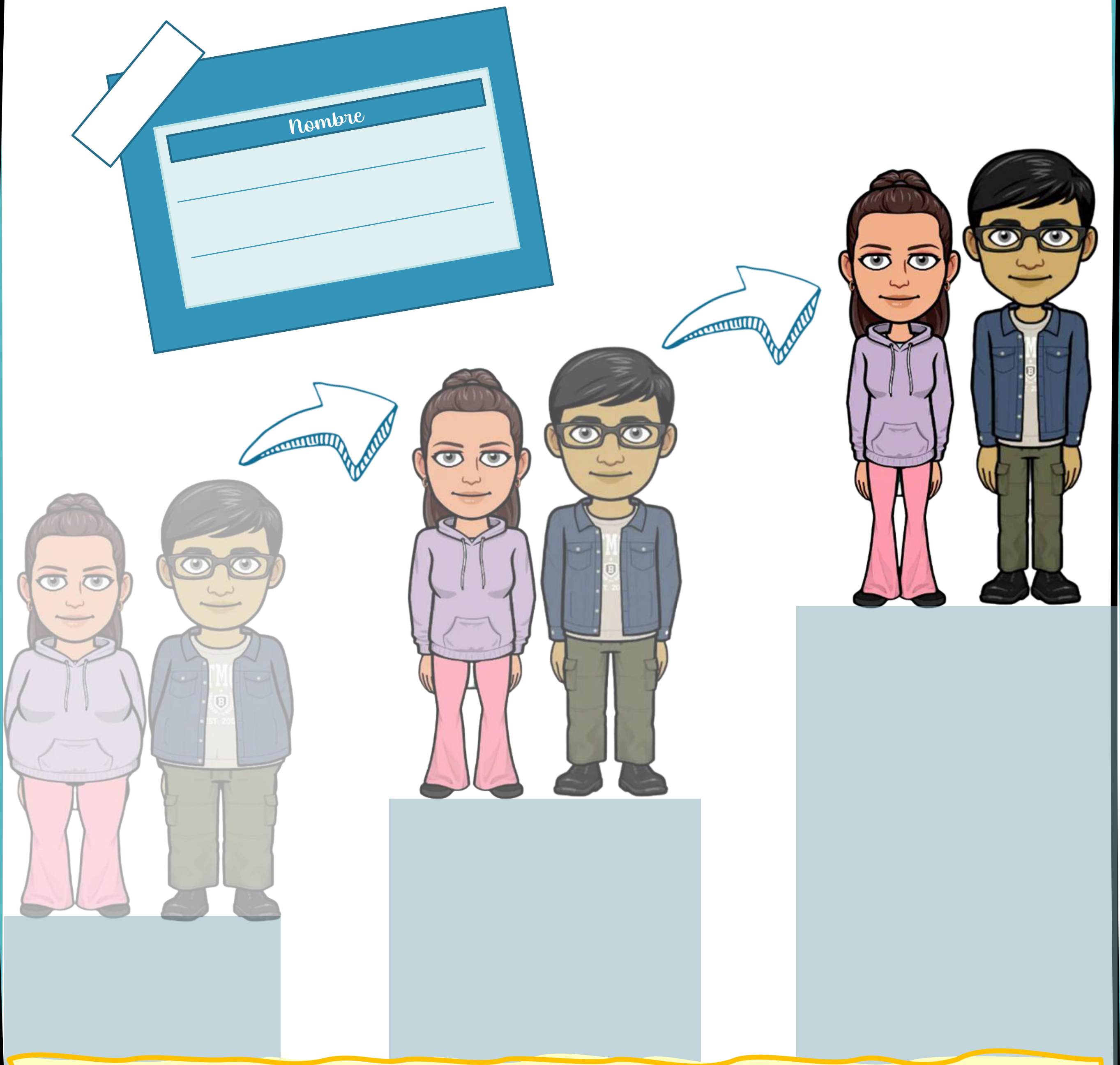

Basado en Grupo de equilibrio™ de estilo de vida. Copyright © 2011 by the University of Pittsburgh

Equilibra tu Estilo de Vida

UTPL

Transforma tu vida con cambios diarios

Su nombre:

Su Estilo de Vida

Información de contacto:
Dirección de trabajo:

Edad _____ años
Teléfono:

E-mail:

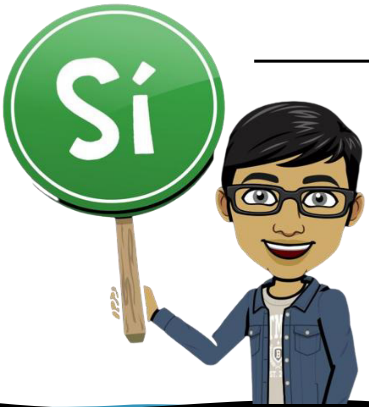

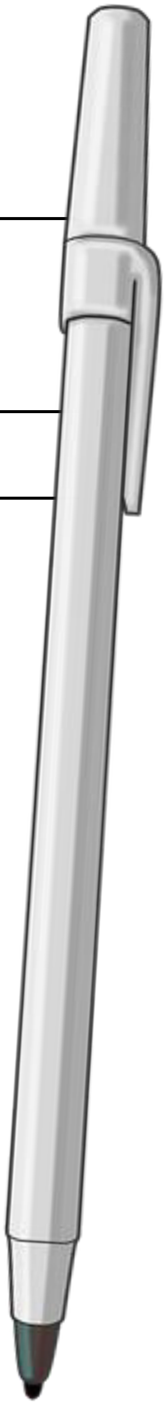

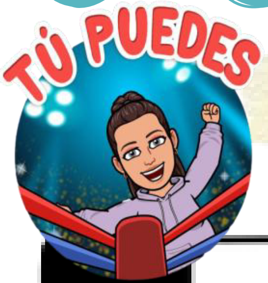

Escriba su propósito

¿Por qué se unió a nuestro programa (Transforma tu vida con cambios diarios)?

¿Qué espero lograr al participar en Transforma tu vida con cambios diarios?

Cómo me ayudará a mí y a otros, el comer sano y mantenerme activo/a:

UTPL

Objetivo:

•Ayudarle a hacer cambios de estilo de vida para prevenir tanto la diabetes como el síndrome metabólico y a su vez las enfermedades cardiovasculares.

Sus metas de Transforma tu vida con cambios diarios serán:

1. Perder peso a través de una alimentación sana.
2. Estar más activo/a físicamente.

Un importante estudio demostró que se puede prevenir la diabetes con una combinación sana y a largo plazo de alimentación balanceada e incremento de la actividad física .

Herman, W. H. (2015). The cost-effectiveness of diabetes prevention: results from the Diabetes Prevention Program and the Diabetes Prevention Program Outcomes Study. *Clinical diabetes and endocrinology*, *1*(1), 1-10.

En este programa le ayudaremos a:

•Aprender sobre alimentación sana y cómo mantenerse activo/a.

•Canalizar lo que le impide comer sano y mantenerse activo/a.

•Modificar su estilo de vida para evitar enfermedades

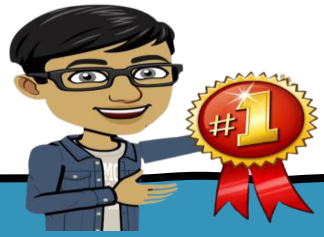

Trabajaremos en EQUIPO

Vamos a contar con usted para:

• Asistir o ver las sesiones de asesoría y seguir las instrucciones del presente folleto.
• Hacer todo lo posible para alcanzar sus metas de alimentación y actividad física. Esto incluye hacer las actividades asignadas para practicar lo que está aprendiendo.
• Llevar un registro de su alimentación y actividad los 7 días de la semana. Sea honesto/a.
• Llevar el registro de su peso.
• Mantenerse dispuesto al cambio.
“Animo, ¡Lo puede lograr!”

Puede contar con nosotros para:

• Proporcionarle información precisa sobre alimentación sana, actividad física y pérdida de peso.
• Contestar sus preguntas.
• Creer que puede alcanzar sus metas de alimentación y actividad física.

Estamos de acuerdo en trabajar juntos en la forma descrita anteriormente.

Firmado: ______________________________

Fecha: _________________

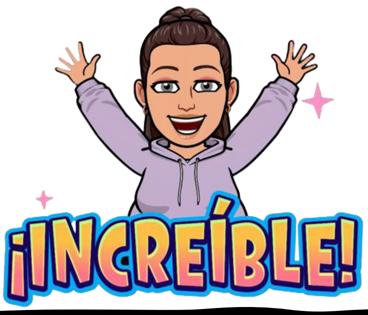

Sesiones de "Transforma tu vida con cambios diarios"

1. Bienvenido a Transforma tu vida con cambios diarios.
2. Buenos Alimentos, Buena Vida
3. Activa tu cuerpo, Activa tu mente
4. Descubre tu motivación interna
5. Equilibra tus calorías, equilibra tu vida
6. Sé más fuerte que tus excusas: Ejercicios para mantener una vida activa
7. La clave es la consistencia: Cómo permanecer
8. Disfruta cada bocado: Consejos para comer conscientemente y controlar la porción
9. Mantén el equilibrio: Consejos para un estilo de vida saludable y sostenible a largo plazo
10. No te rindas: Cómo perseverar cuando la motivación comienza a disminuir

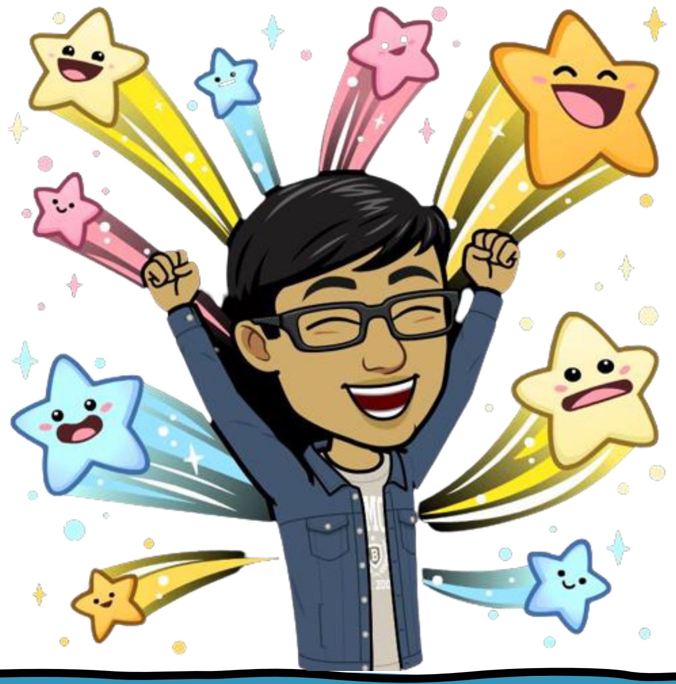

UTPL

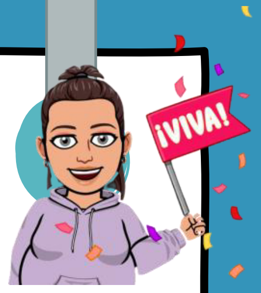

TAREAS SEMANALES

Mes inicio: ___________________Año inicio:________

	Fecha	Peso	Tipo de actividad física diaria y el tiempo dedicado	Cambios en la alimentación	Reflexión y Estado de ánimo
SESION 1					
SESION 2					
SESION 3					
SESION 4					
SESION 5					
SESION 6					
SESION 7					
SESION 8					
SESION 9					
SESION 10					
MEDICION FINAL					

Sesión 1:

Bienvenidos a Transforma tu vida con cambios diarios

Sea un Buen Miembro del Grupo
Un grupo es como un equipo de deportes.

Al trabajar en conjunto, los miembros del grupo pueden:

- Apoyarse y alentarse mutuamente.
- Compartir ideas para resolver problemas
- Motivarse mutuamente.

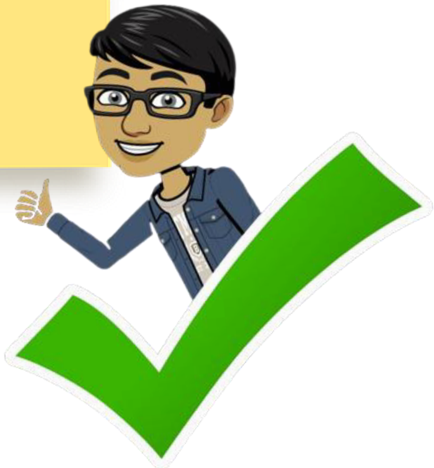

Diez Formas de ser un Buen Miembro/a del Grupo

1. Haga su mejor esfuerzo por asistir a cada convocatoria. Esto es importante, incluso cuando le sea difícil seguir el programa

2. Sea puntual. Avise si no puede asistir.

3. Complete las tareas que debe hacer en casa.

4. Traiga este folleto a cada encuentro.

5. Participe y comparta sus ideas con otros compañeros de trabajo.

7. Comparta lo que le ha funcionado a usted.

8- Revise y vea los videos enviados al grupo de whatsapp

9- Revise siempre el material educativo que se le envía.

10.Haga hincapié en las cosas buenas que ha logrado.

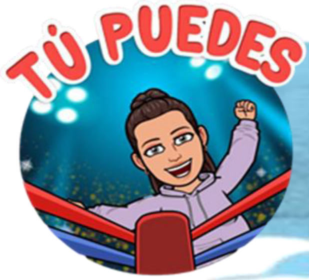

Sesión 2:

Buenos Alimentos, Buena Vida

UTPL

Información básica sobre medidas caseras:

Medida	Abreviatura	Cantidad	Equivalente
Taza	t o tz.	250 gramos	8 onzas<br>16 cucharaditas<br>Un puño
Cucharada	C o cda.	15 gramos	3 cucharaditas<br>La punta del pulgar
Cucharadita	c o cdta.	5 gramos	Punta del dedo índice
Onza	Oz.	28 gramos	Palma de la mano ahuecada
Gramo	g.	1 gramo	El peso de un sujetapapeles

EJERCICIO

Anote los alimentos que comería en un día para cada grupo de alimentos que sugiere el plato del buen comer.

	Desayuno	Almuerzo	Cena	Refrigerios
Cereales				
Verduras				
Frutas				
Proteínas				

Sesión 2:
Buenos Alimentos, Buena Vida

El plato del buen comer

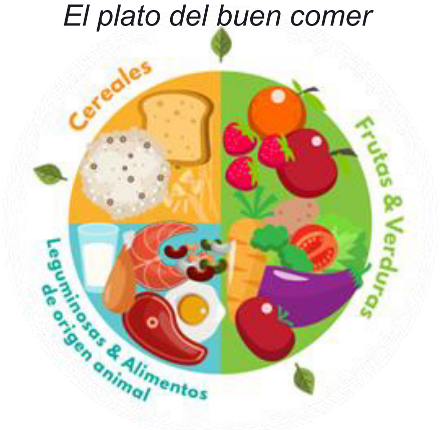

•Cereales: Por lo menos la mitad de los cereales que consumas deben ser integrales.
•Vegetales: Varía y combina las verduras.
•Frutas: Consume frutas como postre.
•Proteínas: Prefiere proteínas bajas en grasa como las carnes blancas.

Información sobre alimentos donde se pueden encontrar grasas saludables

**Grasas saludables**	Aguacate
	Nueces y Almendras
	Semillas de girasol
	Aceites vegetales (oliva, girasol, canola, maíz, soya)
	Pescados grasos (salmón, atún, sardinas, truchas,etc)
	Cacao
	Aceitunas
	Yema del huevo

Actividad: Cuestionario sobre grasas buenas y malas

¿Qué son las grasas buenas y por qué son importantes para el cuerpo?

____

____

_

Menciona tres ejemplos de alimentos que contengan grasas buenas.

____

____

____

¿Cuál es la principal diferencia entre las grasas saturadas y las grasas insaturadas?

____

____

_

Sesión 3:

Activa tu cuerpo, Activa tu mente

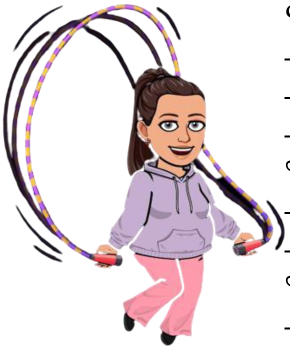

¿Qué tan activo/a es en la actualidad? (Tipo de actividades que hace, con quien, con qué frecuencia y por cuánto tiempo)

____

____

____

¿Hay actividades que hacía en el pasado que no hace en la actualidad?

____

____

¿Por qué dejó de hacerla?

____

____

____

Meta: Estar activo/a por ____________________minutos esta semana.

	Lo que haré	Cuándo (horario)	Minutos
Lunes			
Martes			
Miércoles			
Jueves			
Viernes			
Sábado			
Domingo			

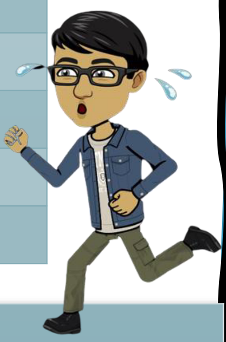

LO HICE							
L	L	L	L	L	L	L	L
M	M	M	M	M	M	M	M
M	M	M	M	M	M	M	M
J	J	J	J	J	J	J	J
V	V	V	V	V	V	V	V
S	S	S	S	S	S	S	S
D	D	D	d	D	D	D	D

Sesión 3:
Activa tu cuerpo, Activa tu mente

¿Dónde debo comenzar? Durante la semana pasada, ¿Cuántos minutos de actividad física piensa que hizo? Si es menos de 60 minutos, su objetivo para la próxima semana es 60 minutos. Si hizo más de 60 minutos la semana pasada, haga la misma cantidad esta semana y anote su actividad.

Ejemplo

Semana 4:
Camine 60 minutos por semana
(12 minutos en 5 días por semana).
Semana 5:
Camine 90 minutos por semana
(18 minutos en 5 días por semana).
Semana 6:
Camine 120 minutos por semana
(24 minutos en 5 días por semana).
Semana 7+:
Camine 150 minutos por semana
(30 minutos en 5 días por semana).

Lo que significa las siglas R.I.C.E. :	Lo que debe hacer:
**R-reposo (limite el movimiento)**	• Suspenda la actividad.<br>• Descanse unos días. Esto prevendrá un sangrado profuso<br>• En ocasiones se necesita entablillar, esparadrapo o vendas
**I-coloque hielo**	• Aplique hielo o compresas frías durante las primeras 24 a 36 horas después de la lesión. Esto reduce el dolor el sangrado y la hinchazón.<br>• Aplique por 10 minutos y descanse por otros 10 minutos.<br>• ***Siempre*** envuelva el hielo o la compresa en una toalla o paño. Aplicarlo directamente o envuelto en plástico puede causar congelación y más lesión.
**Compresión (Presión)**	• Aplique presión envolviendo la lesión con una venda elástica. Esto ayuda a reducir la inflamación y el flujo de sangre a el área.<br>• La venda debe ser suficientemente tensa para reducir el flujo de sangre pero no para cortarlo completamente. Afloje la venda si sus dedos se adormecen o pierden su color.
**Eleve**	• Levante el área lesionada arriba del nivel del corazón. Manténgala elevada cuanto le sea posible, no solo cuando aplique hielo.<br>• Esto ayuda a reducir la hemorragia interna y la acumulación de sangre que puede causar dolor punzante.

Si hay Dolor…

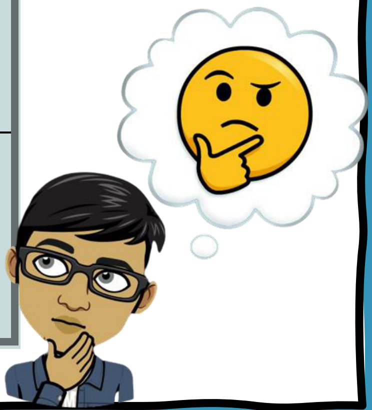

Sesión 3:
Activa tu cuerpo, Activa tu mente

Google Fit

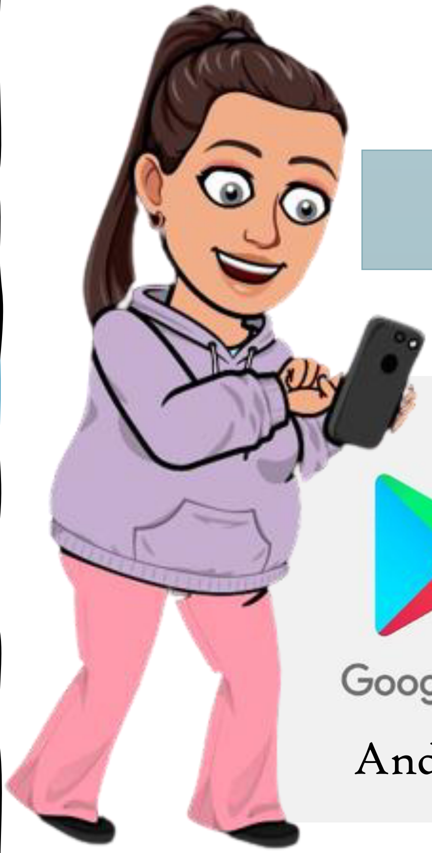

Cómo descargar

Diríjase a:

Google Play
Android

App Store
IOS

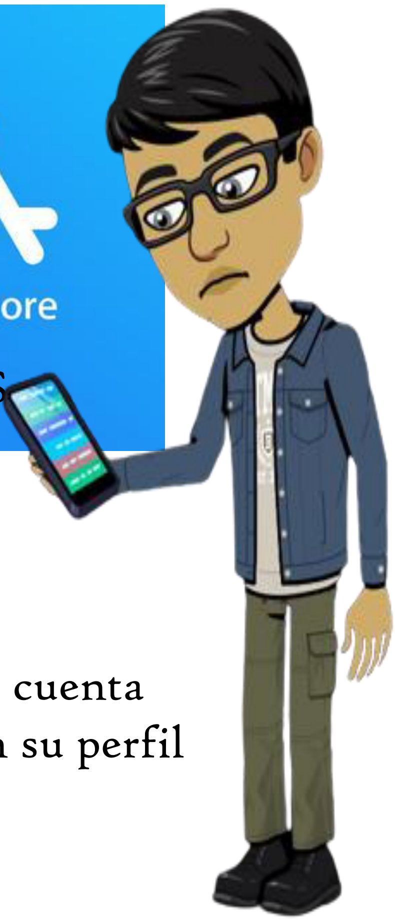

Buscar: Google Fit

1. Descargar
2. Registrarse con el correo o colocar su cuenta
3. Elegir el objetivo diario, ingresando en su perfil

Sesión 3:
Activa tu cuerpo, Activa tu mente

Combinación de actividades

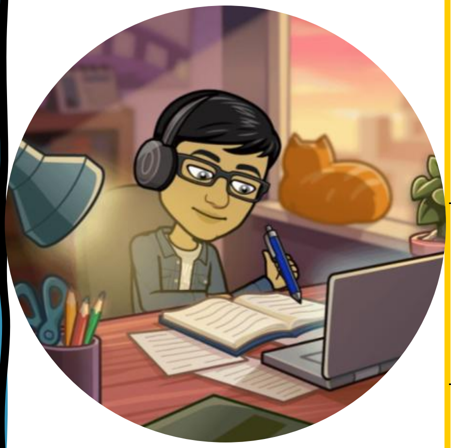

**Maneras de Variarlo:**	**Ejemplos:**
Agregue Variedad:<br>• Haga algo nuevo<br>• Haga la misma actividad en un lugar u horario diferente<br>• Manténgase activo/a como una nueva forma de socializar. Comparta su actividad con alguien nuevo	
Diviértase mientras esta activo/a<br>• Baile<br>• Escuche música o libros en cinta mientras está activo/a<br>• Participe en eventos activos como hacer recorrido turístico a pie o únase a un grupo de ciclismo	
Retese<br>• Trate de agregar más pasos a su día<br>• Entrénese para un evento como un maratón de caminata o una haga una excursión que le rete.	

Día 1: Día Normal	Día 2: Día de Estilo de Vida
Total # de pasos= _____	Total # de pasos= _______

	REGISTRO DE PASOS														
L		L		L		L		L		L		L		L	
M		M		M		M		M		M		M		M	
M		M		M		M		M		M		M		M	
J		J		J		J		J		J		J		J	
V		V		V		V		V		V		V		V	
S		S		S		S		S		S		S		S	
D		D		D		d		D		D		D		D	

Sesión 4:
Descubre tu motivación interna

1. Mantenga fuera de su casa y oficina alimentos altos en grasas/calorías. O manténgalos donde no los pueda ver. **Fuera de la vista, fuera de la mente.**
2. Mantenga opciones bajas en grasas/calorías donde las pueda ver, alcanzar y comer fácilmente. Ejemplos: **Frutas frescas, vegetales crudos (ya lavados y preparados), bebidas bajo en azúcar, gelatina sin azúcar, paletas de agua sin azúcar.**
3. **Límite donde come a un solo sitio.**
4. **Cuando coma, limite otras actividades**

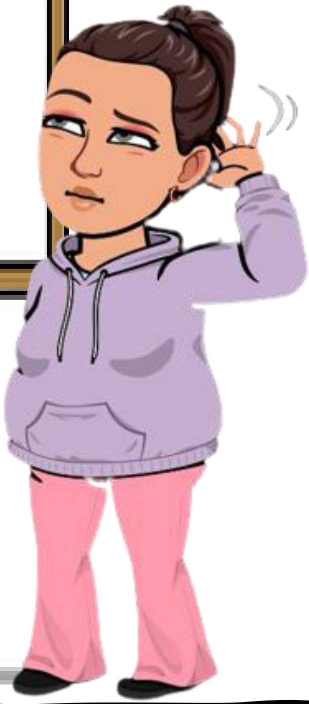

Sesión 4:
Descubre tu motivación interna

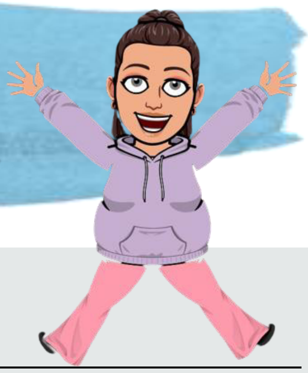

Voy a deshacerme de una de las señales problemáticas de alimentos.
¿De qué señal problemática de alimentos se va a deshacer?

____

¿Qué necesita hacer para deshacerse de ella?

____

¿Qué problemas podría tener para resolverlo ? ¿Qué hará para resolverlo?

____

Agregar una señal positiva para mantenerse más activo/a. ¿Qué señal de actividad va a agregar?

____

¿Qué necesitará hacer para poder agregarla?

____

¿Qué problemas podría tener? ¿Qué hará para resolverlo?

____

Planificar qué me gustaría hacer

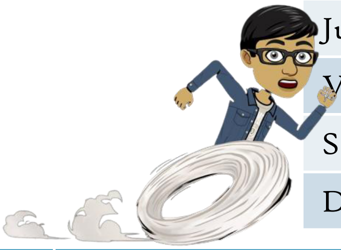

	Lo que haré	Cuando	Minutos
Lunes			
Martes			
Miércoles			
Jueves			
Viernes			
Sábado			
Domingo			

	LO LOGRE														
L		L		L		L		L		L		L		L	
M		M		M		M		M		M		M		M	
M		M		M		M		M		M		M		M	
J		J		J		J		J		J		J		J	
V		V		V		V		V		V		V		V	
S		S		S		S		S		S		S		S	
D		D		D		d		D		D		D		D	

Sesión 4:
Descubre tu motivación interna

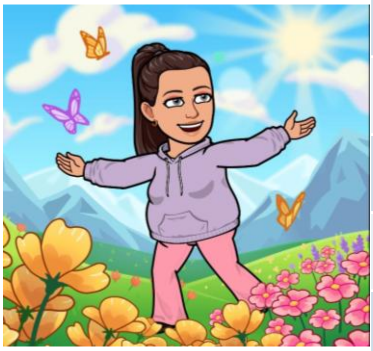

Cadena de acción LAZOS	¿Qué opciones tengo?

Buena alimentación

Voy a ... ______________________________

¿Cuándo? ... ______________________________

Voy a hacer esto primero______________________________

Obstáculos que podrían presentarse: ______________________________

Como los voy a resolver: ______________________________

Voy a hacer lo siguiente para que mi éxito sea más probable: ______________________________

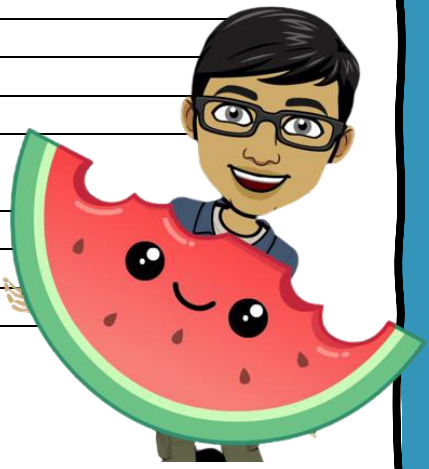

Sesión 4:
Descubre su motivación interna

Actividad Física

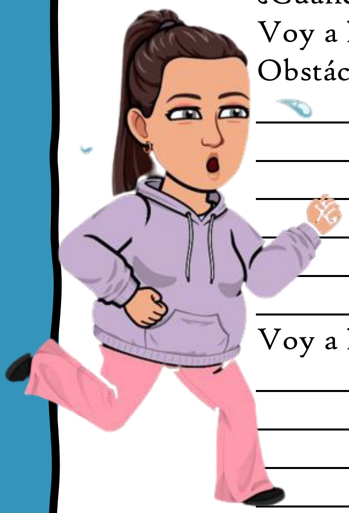

Voy a ... ______

¿Cuándo? ... ______

Voy a hacer esto primero______

Obstáculos que podrían presentarse:	Como los voy a resolver:

Voy a hacer lo siguiente para que mi éxito sea más probable:

Hábito Saludable:
motivación

Voy a ... ______

¿Cuándo? ... ______

Voy a hacer esto primero______

Obstáculos que podrían presentarse:	Como los voy a resolver:

Voy a hacer lo siguiente para que mi éxito sea más probable:

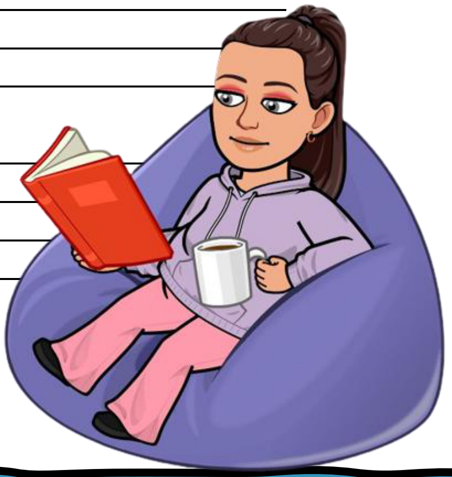

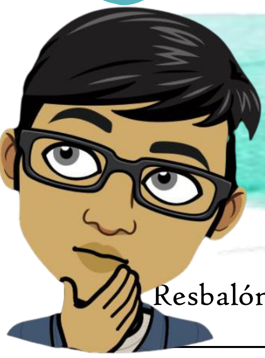

Sesión 4:
Descubre su motivación interna

Resbalón

Duendes Mentales:

1. A continuación escriba ejemplos de pensamientos negativos o contraproducentes.
2. Repita cada pensamiento en alto, luego diga, "¡ALTO!"
3. Responda, nuevamente en voz alta con un pensamiento positivo. Anótelo a continuación.

Pensamiento Negativo	¡ALTO!	Pensamiento Positivo
	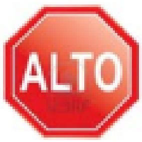	
	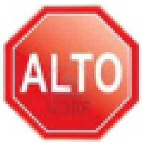	
	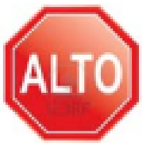	
	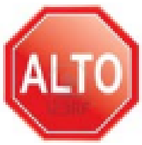	
	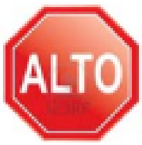	

Sesión 4:
Descubre su motivación interna

Los resbalones no impiden su progreso.
Lo que afecta su progreso es la forma en la que reacciona ante esos resbalones.

Después de un resbalón:

1. **Responda a los pensamientos negativos con pensamientos positivos.** Los pensamientos negativos son su peor enemigo. Respóndales. "No soy un fracaso porque tuve un resbalón. Me puedo recuperar y continuar intentándolo".
2. **Pregúntese que sucedió.** Aprenda de sus resbalones. ¿Lo podría evitar en el futuro? ¿Podría controlarlo mejor?
3. **Recupere el control lo antes posible.** No se diga a sí mismo/a, "Ya arruiné todo este día." Haga que su siguiente comida sea sana. Vuelva a su horario de actividad física lo antes posible.
4. **Hable con alguien que le preste apoyo.** Llame a su consejero/a de estilo de vida o alguna amistad. Platique de su nueva estrategia para responder a los resbalones. Comprométase a renovar su esfuerzo.
5. **Enfóquese en todos los cambios positivos que ha logrado.** Está haciendo cambios de por vida. Los resbalones son parte del proceso.

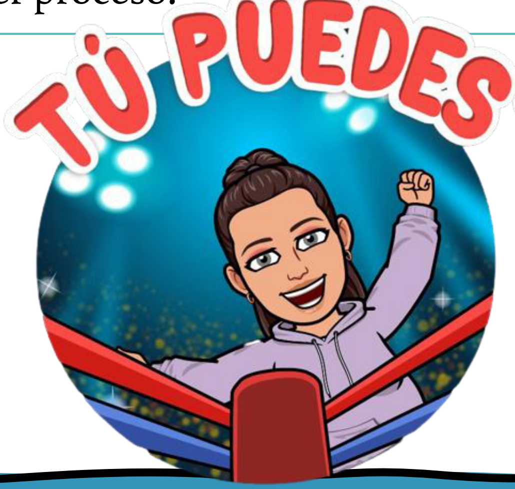

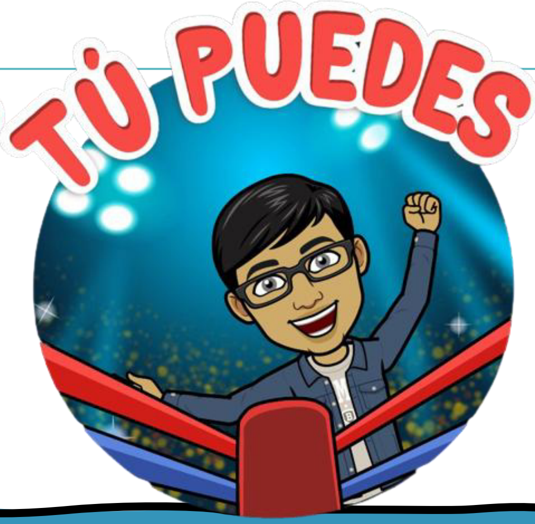

Sesión 5:
Equilibra tus calorías, equilibra tu vida

Calorías de alimentos – calorías de actividad

	Calorías de alimentos
1. Su peso puede permanecer igual<br><br>Las calorías ingeridas (alimentos) son igual a las calorías utilizadas al momento de realizar actividad física.	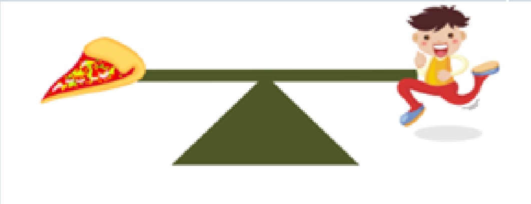
2. Usted puede aumentar de peso<br><br>Las calorías ingeridas son más que las calorías utilizadas al momento de realizar actividad física.	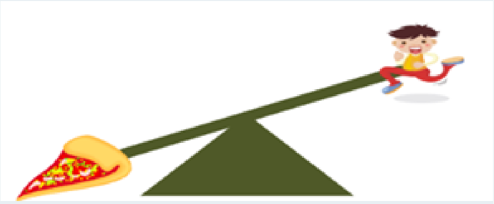
3. Usted puede bajar de peso.<br><br>Las calorías ingeridas son menos que las calorías utilizadas al momento de realizar actividad física.	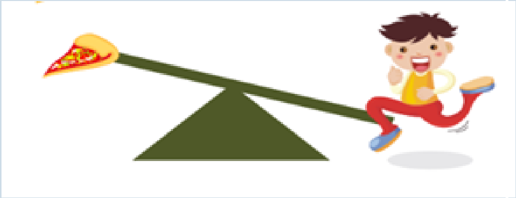
4. Puede alcanzar un nuevo equilibrio en un nuevo peso.<br><br>Las calorías incorporadas son las mismas que las calorías utilizadas al momento de realizar actividad física.	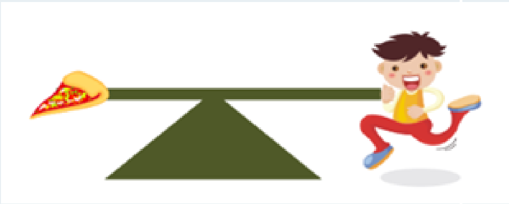

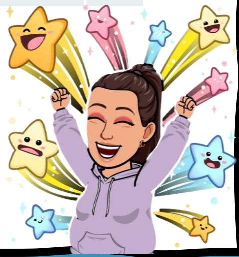

Sesión 5:
Equilibra tus calorías, equilibra tu vida

Actividad:

Elige el alimento que consumes con mayor frecuencia

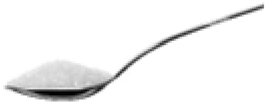

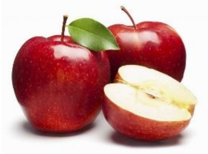

La fruta nos aporta azúcar natural pero también agua, vitaminas, minerales y fibra, y tiene grandes beneficios para el cuerpo, por esto la fruta es la mejor opción.

¿Por qué de tu elección?

____

____

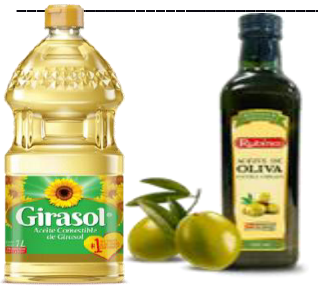

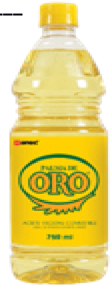

El consumo habitual de alimentos con grasas saturadas tiene repercusiones en el organismo, esto incluye el aceite de palma, por lo que se recomienda el consumo de aceite de oliva – girasol para una vida más saludable.

¿Por qué de tu elección?

____

____

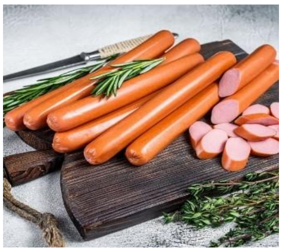

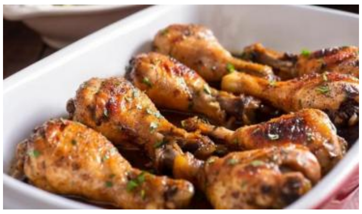

No consumir alimentos procesados con elevado porcentaje de sal y grasa. Preferir el pollo, una proteína saludable.

¿Por qué de tu elección?

____

____

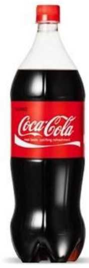

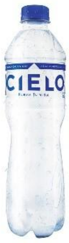

Al consumir un vaso de coca cola, estamos consumiendo un aproximado de 10 cucharadas de azúcar al mismo tiempo. Sin duda alguna, el agua es lo que nuestro organismo realmente necesita.

¿Por qué de tu elección?

____

____

Sesión 6:
Fortalezca su programa de Ejercicios

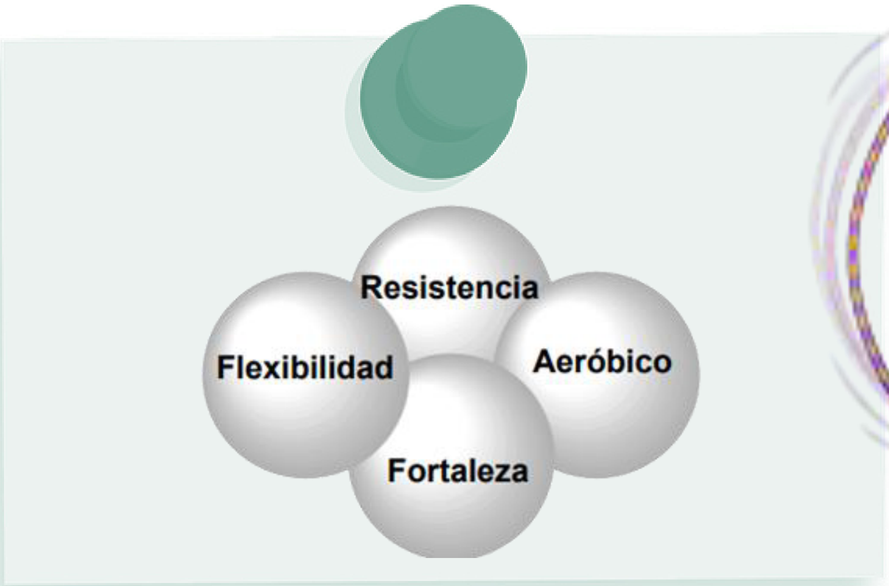

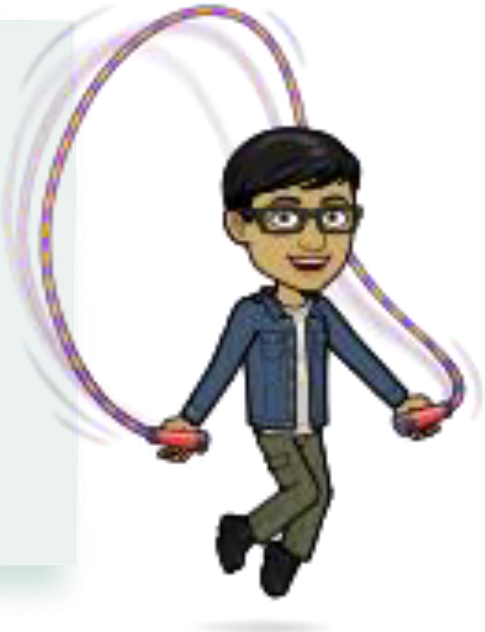

Lo que debe hacer:

Mantenga el registro de su peso.

- Pésese en su casa todos los días o por lo menos una vez por semana a la misma hora del día.
- Anote su peso en un registro personal
- Mantenga el registro de lo que come y bebe
- Mantenga el registro de su actividad física en registro personal
- Manténgase físicamente activo por lo menos 150 minutos por semana

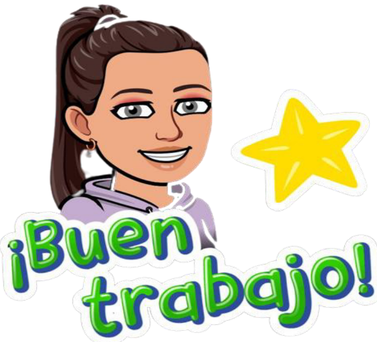

Sesión 6:
Sé más fuerte que tus excusas: Ejercicios para mantener una vida activa

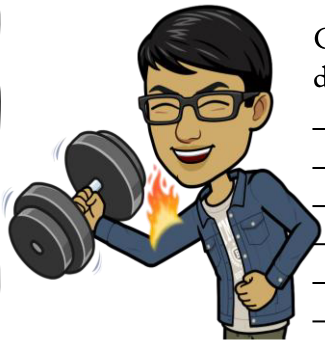

Con lo explicado en las sesiones y con apoyo del material educativo describa las modificaciones en su rutina:

____

____

____

____

____

____

- Elija una actividad dentro del entrenamiento de resistencia.
  Incluya actividades de flexibilidad en su programa de ejercicio.

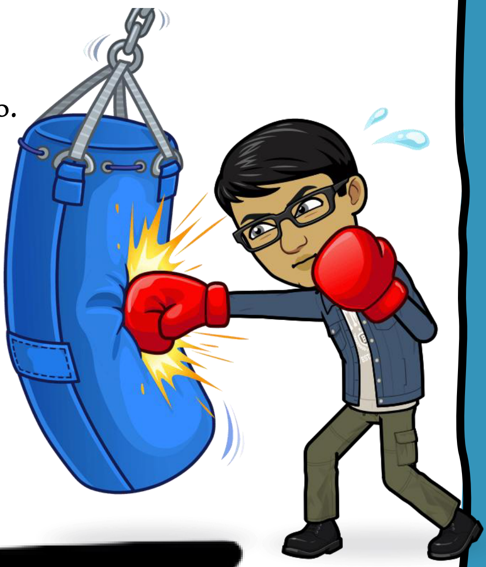

Notas adicionales

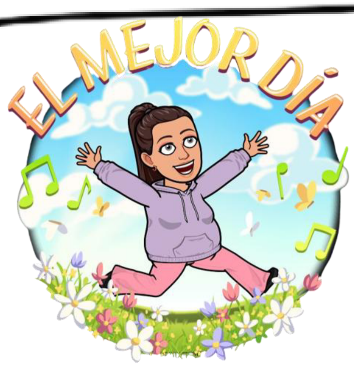

Sesión 6:
Sé más fuerte que tus excusas: Ejercicios para mantener una vida activa

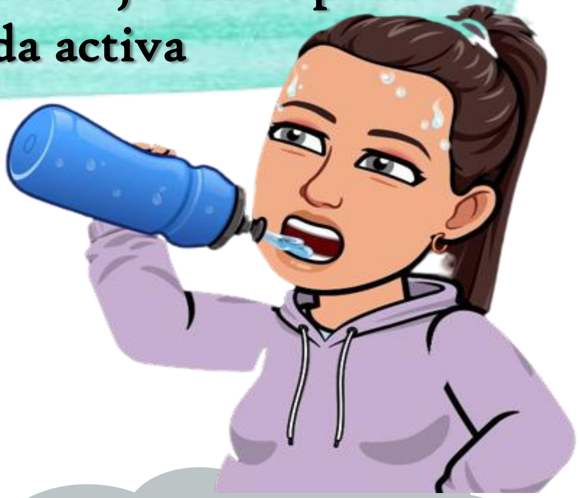

SIN DOLOR SI HAY GANANCIA ¡Escuche a su cuerpo!

El calentamiento relaja los músculos y previene lesiones.

Asegúrese de estirar ambos lados del cuerpo - Manténgase en equilibrio

NO contenga la respiración

Mantenga el estiramiento firme por 15 a 30 segundos. NO rebote

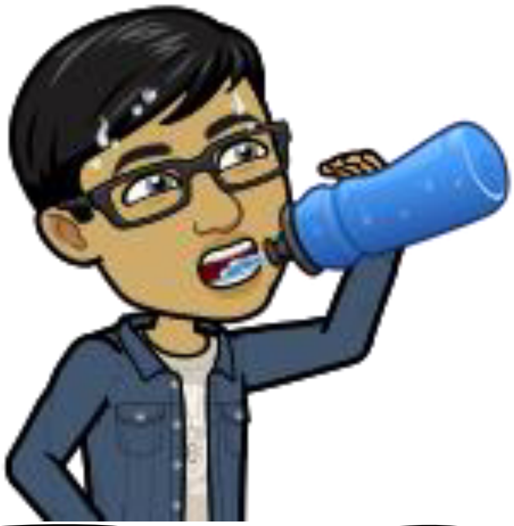

Sesión 7:
La clave es la consistencia: Cómo permanecer

UTPL

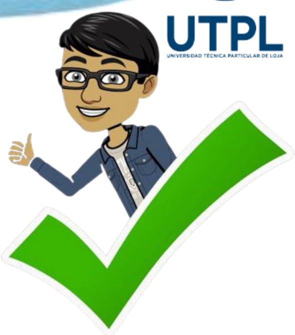

Señales sociales

Haga que las señales sociales trabajen a su favor: escriba ejemplos de situaciones que fortalecen su cambio al estilo de vida.

Señales sociales útiles:	Ejemplos:
Ver a otras personas que hacen actividad física o comen alimentos sanos.	
Le ofrecen alimentos sanos o le invitan a hacer algo activo.	
Le felicitan.	
Le dan cumplidos.	

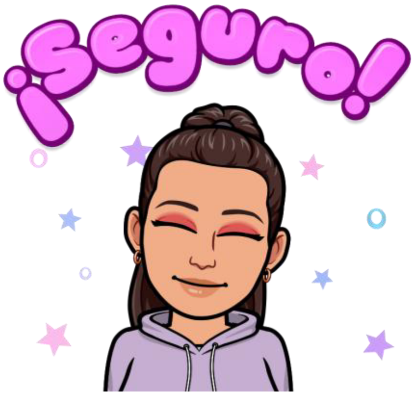

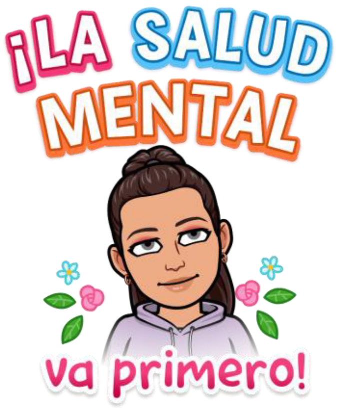

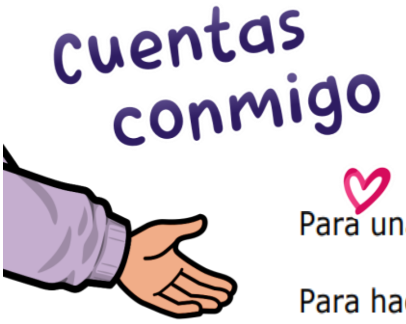

¿Quién puede darle apoyo?

Defina quien puede ayudarlo en su cambio de estilo de vida.

Para una alimentación sana: ____________________________________

Para hacer actividad física: ____________________________________

Sesión 7:
La clave es la consistencia: Cómo permanecer

Haga un plan de acción sobre los factores sociales, como ya se ha enseñado anteriormente:

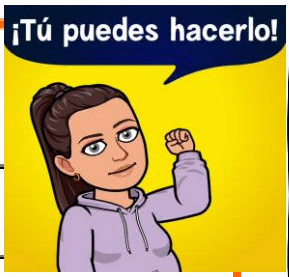

Haga un plan de acción positivo.

Voy a: ____

¿Cuándo? ____

Voy hacer esto primero: ______________________________

Obstáculos que pueden presentarse: Como los voy a resolver:

______________________________ ______________________________

______________________________ ______________________________

______________________________ ______________________________

Voy a hacer lo siguiente para que mi éxito sea más probable:______

____

____

¿Cómo podemos ayudarle?____

____

____

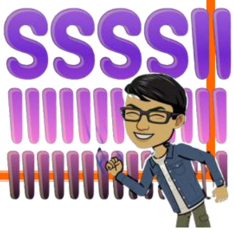

Sesión 7:
La clave es la consistencia: Cómo permanecer

Haga un plan de acción sobre la motivación, como ya se ha enseñado anteriormente:

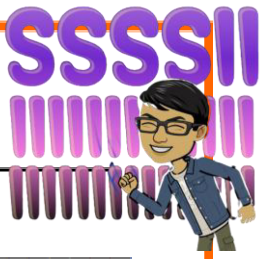

Haga un plan de acción positivo.

Voy a: ____

¿Cuándo? ____

Voy hacer esto primero: ______________________________

Obstáculos que pueden presentarse: Como los voy a resolver:

______________________________ ______________________________

______________________________ ______________________________

______________________________ ______________________________

Voy a hacer lo siguiente para que mi éxito sea más probable:_____

____

____

¿Cómo podemos ayudarle?______________________________________

____

____

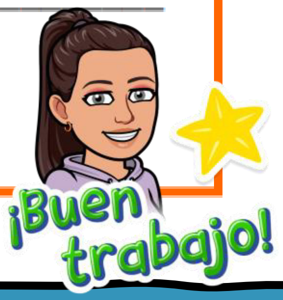

Sesión 7:
La clave es la consistencia: Cómo permanecer

1. Manténgase consciente de los beneficios que ha logrado y espera lograr.

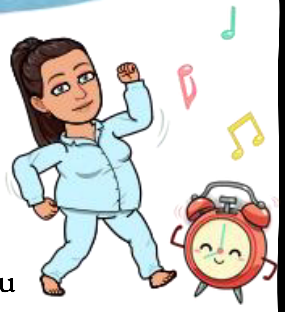

¿Qué es lo que esperaba lograr cuando comenzó en Transforma tu vida con cambios diarios? ¿Ha logrado alcanzar estas metas?

2. Reconozca sus éxitos

¿De qué cambios en su alimentación y actividad se sientes más orgulloso/a?

3. Control del estrés

El estrés es tensión o presión. Muchas personas reaccionan al estrés comiendo de más y estando inactivas. ¿Qué tipo de situaciones le causan estrés?

¿Qué es lo que siente cuando está estresado/a?

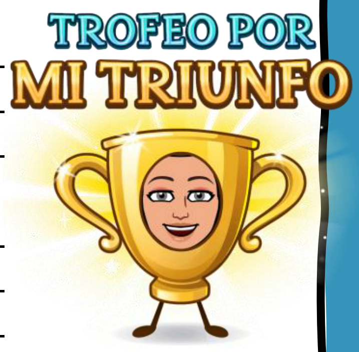

Sesión 7:
La clave es la consistencia: Cómo permanecer

Revisión del progreso

Los cambios que ha hecho para estar más activo/a:

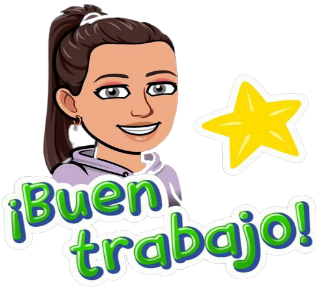

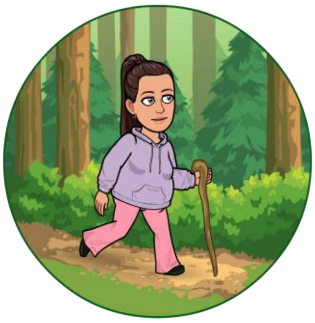

Los cambios que ha realizado para comer menos grasa (y menos calorías):

¿Ha logrado alcanzar su meta de peso? ☐ Si ☐ No
¿Ha logrado alcanzar su meta de actividad física? ☐ Si ☐ No

Si la respuesta es negativa, ¿Qué puede hacer para mejorar su progreso?

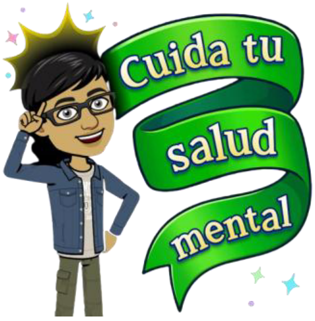

Sesión 7:
La clave es la consistencia: Cómo permanecer

Voy a:

- Llevar el registro de mi peso, consumo de alimentos y actividad física.
- Continuar llevando el registro de mis minutos de actividad.
- Tratar de hacer tres de las cosas que disfruto que están anotadas en esta página pero que raramente hago.

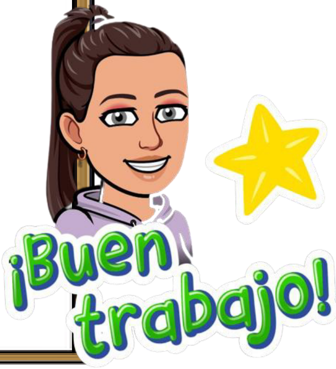

Elija cosas que no están relacionadas con alimentos que son fáciles de hacer.

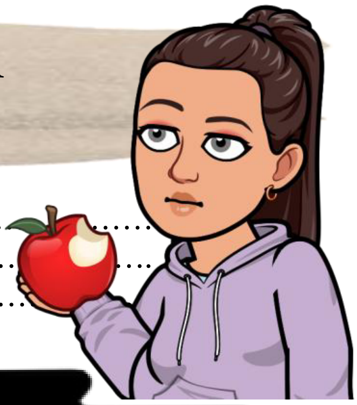

1. ....
2. ....
3. ....

Programe cómo incorporar estas actividades placenteras en su vida.

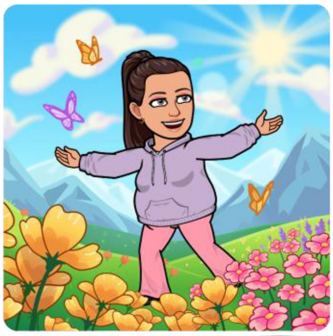

Sesión 8:
Disfruta cada bocado: Consejos para comer conscientemente y controlar la porción

Actividades

¿Qué alimentos con alto contenido de grasas come?

Un ejemplo de menú de almuerzo

Alimento	Cantidad adivinada	Cantidad real	Gramos de grasa	Calorías

Preguntas:

¿Qué estrategias utiliza para elegir opciones saludables cuando come fuera de casa?

____

____

¿Sabe cómo controlar las porciones cuando come en restaurantes?

____

____

¿Sabe cómo equilibrar sus comidas cuando tiene que comer fuera de casa frecuentemente?

____

____

¿Presta atención a los ingredientes utilizados en los platos que consume fuera de casa?

____

____

¿Conoce la importancia de consumir una variedad de colores en frutas y verduras para obtener diferentes nutrientes?

____

____

Sesión 8 :
Disfruta cada bocado: Consejos para comer conscientemente y controlar la porción

Elige opciones verdes, tu salud lo agradece, verduras y ensaladas, tu cuerpo se fortalece.

No te saltes comidas, al hambre no engañes, alimentación regular, tus energías mantén.

Come despacio, saborea cada bocado, escucha a tu cuerpo, la saciedad encontrarás.

Investiga el menú, antes de entrar al lugar, opciones saludables, hay que buscar

Platos a la parrilla, opción más sana, evita lo frito, y tu dieta se afina

Sesión 9:

Mantén el equilibrio: Consejos para un estilo de vida saludable y sostenible a largo plazo

- Enumere las 5 razones principales que son de mayor importancia para usted

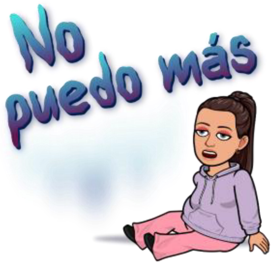

Razones por las Que Quiero Perder Peso (y Mantener la Pérdida de Peso)	
Quiero mirarme en el espejo y sentirme bien.	
Quiero lucir mejor ante los demás.	
Quiero poder usar una talla más pequeña.	
Quiero poder salir a comprar ropa con menos dificultad.	
Quiero dejar de pensar en cómo me ven los demás.	
Quiero ser felicitado(a) por mi apariencia.	
Quiero prevenir dolencias físicas y enfermedades.	
Quiero estar cómodamente activo(a).	
Quiero tener una vida mas larga.	
Quiero tener más energía	
Quiero estar en buen estado físico.	
Quiero levantarme en la mañana y sentirme más saludable.	
Quiero sentirme mejor conmigo mismo(a).	
Quiero sentirme más en control de mi vida.	
Quiero sentirme que he logrado algo muy importante.	
Quiero sentir confianza en mí mismo(a)	
Quiero dejar de tener pensamientos negativos sobre mí mismo(a)	
Quiero sentirme más feliz en ambientes sociales.	
Quiero hacer más y diferentes tipos de actividades.	
Quiero que mi familia se sienta orgullosa de mí.	
Quiero expresar lo que quiero con seguridad y confianza.	
Quiero salir a comer con otros y sentirme cómodo(a).	
Quiero que me dejen de molestar sobre mi peso.	
Otra razón:	
Otra razón:	
Otra razón:	

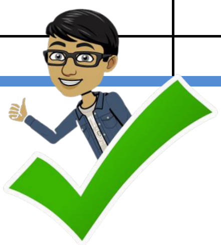

Sesión 9 :

Mantén el equilibrio: Consejos para un estilo de vida saludable y sostenible a largo plazo

Dese Crédito:

Anote 3 cambios positivos logrados durante "Transforma tu vida con cambios diarios", que le han ayudado a manejar su peso o mejorar su salud:

1. ____________________
2. ____________________
3. ____________________

A partir de hoy, anote 1 o 2 cosas que hizo bien cada día, además haga una lista de sus conductas que merecen crédito y anote palabras o frases positivas para felicitarse por sus logros.

Por ejemplo:

No me serví una segunda porción en la fiesta, estoy en control

Hoy caminé 30 minutos…¡Felicidades!

Hoy me pesé

Al conscientemente darse crédito y felicitarse, reforzará su confianza en sí mismo y tomará mayor conciencia de que es fuerte y está en control.

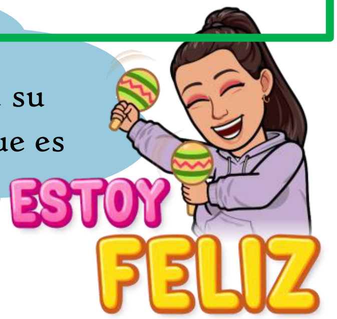

Rétese a sí mismo:

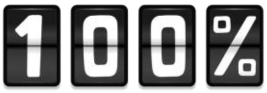

• Practique reemplazar los pensamientos que lo sabotean con pensamientos positivos.

____

____

____

____

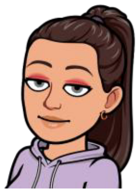

• Anote 1 o 2 cosas que hizo correctamente

____

____

____

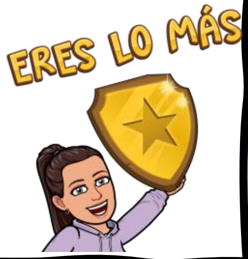

Sesión 9 :
Mantén el equilibrio: Consejos para un estilo de vida saludable y sostenible a largo plazo

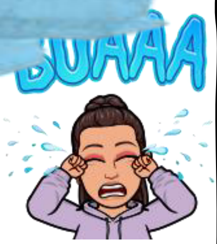

1. ¿Con que frecuencia se siente estresado?

□Frecuentemente □Algunas veces □Casi nunca

2. ¿Cómo sabe cuándo está bajo estrés? Describa su experiencia.

3. Haga un listado de las cosas que le están haciendo sentirse estresado en este momento.

4. ¿Cuáles son algunas de las maneras en las que usted maneja el estrés en su vida?

5. ¿Cuál de estas formas de hacer frente al estrés piensa es negativa o no sana?

6. ¿Cuál de estas formas de hacer frente al estrés piensa es positiva o sana?

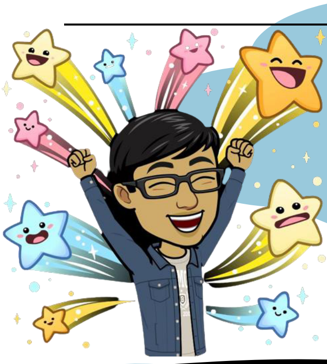

- Practique decir "No." Trate de decir "Si" solo cuando sea importante para usted.

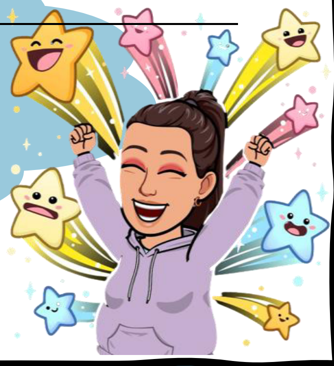

Sesión 9 :
Mantén el equilibrio: Consejos para un estilo de vida saludable y sostenible a largo plazo

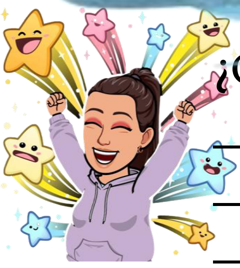

¿Cómo se siente cuando obtiene el sueño adecuado?

¿Cómo se siente cuando duerme muy pocas horas?

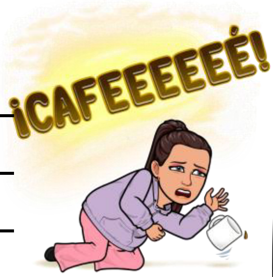

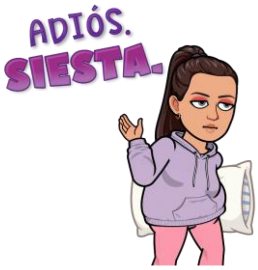

¿Cómo se siente cuando duerme demasiadas horas?

Dormir suficientes horas es un elemento importante para hacer frente al estrés. Esto hace que sea más fácil hacer cambios en la conducta.

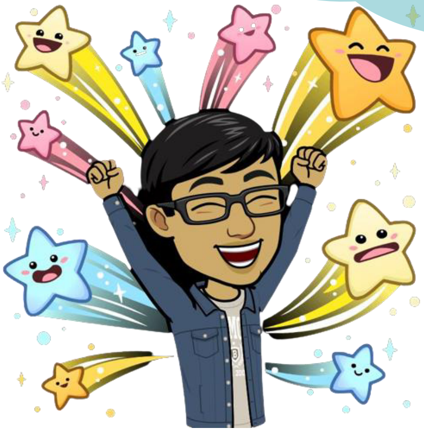

"La risa es como un ejercicio **interno.**"
Norman Cousins

Sesión 9 :
Mantén el equilibrio: Consejos para un estilo de vida saludable y sostenible a largo plazo

Lo Básico:

- Mantenga el registro de peso.
- Pésese en su casa a diario o por lo menos una vez por semana a la misma hora del día.
- Anote su peso en su registro
- Mantenga el registro de sus comidas y bebidas anotándolas en su registro.
- Mantenga el registro de actividad física en su registro.
- Manténgase físicamente activo por lo menos 150 minutos por semana

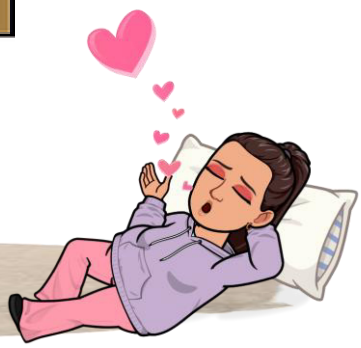

Rétese a sí mismo:

- Practique técnicas de relajación en su casa y/o en su sitio de empleo.

Notas adicionales

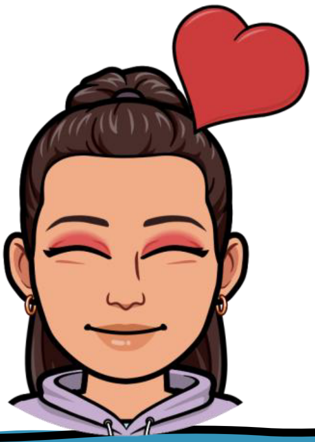

Sesión 10 :

No te rindas: Cómo perseverar cuando la motivación comienza a disminuir

- Escriba en la tabla cuál es su actividad física actual.

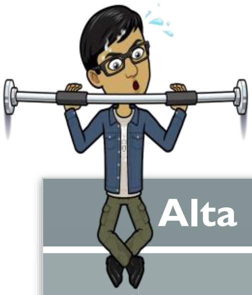

	Alta	Baja
Bajo		
Alta		

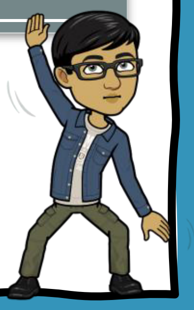

Sesión 10 :
No te rindas: Cómo perseverar cuando la motivación comienza a disminuir

Cuantas horas pasa sentado

	1	2	3	4	5	6	7	8	9	10	11	12
L												
M												
M												
J												
V												
S												
D												

- Piense en las actividades que actualmente realiza; ¿hay algo nuevo que le gustaría intentar?

¿Cuáles son algunas maneras en las que puede reducir la inactividad en su tiempo libre?

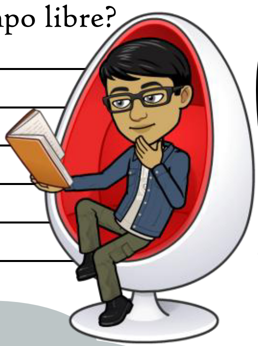

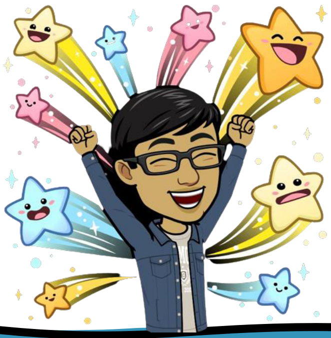

Un estilo de vida sano es su mejor defensa en contra de enfermedades del corazón.

Sesión 10 :
No te rindas: Cómo perseverar cuando la motivación comienza a disminuir

Reflexionar sobre su propia historia de estilo de vida sano.

¿Qué palabras de sabiduría le gustaría compartir con personas a las que recientemente les han informado están a riesgo de padecer diabetes y/o enfermedades cardiovasculares?

¿Qué es lo que resulto más útil cuando se sentía desalentado(a) acerca de su progreso?

Escriba un mensaje a sí mismo(a) para el futuro. Haga una declaración poderosa que le servirá de guía para los próximos meses y años.

Estas conductas son la fundación para tener éxito en su estilo de vida sano.

¡Siga usándolas!

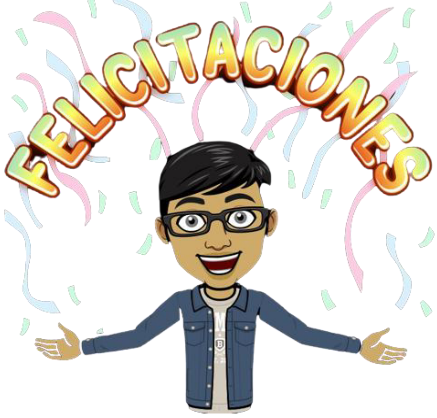

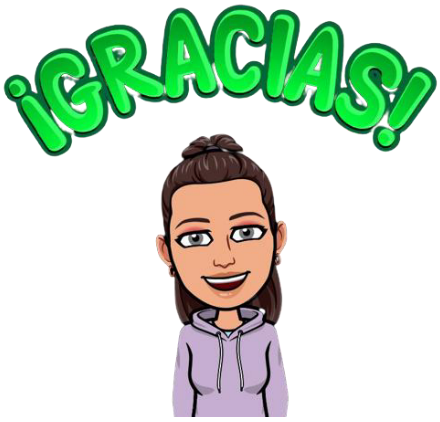

"La felicidad es el resultado de vivir en armonía con nuestro cuerpo, mente y espíritu. Mejorar nuestro estilo de vida es el camino para lograr esa armonía y disfrutar de una vida plena y feliz."

¿CÓMO VOY? LLEVANDO EL REGISTRO DE MI PESO

NOMBRE: ____ META DE PESO: ___________ AÑO: ________

1. EN BLOQUE SUPERIOR CON SOMBRA, ANOTE SU PESO ACTUAL.
2. DEBAJO DEL BLOQUE SUPERIOR CON SOMBRA, ANOTE KG EN ORDEN DESCENDIENTE.
3. DETERMINE META DE PÉRDIDA DE PESO Y HAGA UNA LÍNEA RECTA A TRAVÉS DE LA PAGINA
4. EN EL BLOQUE SUPERIOR CON SOMBRA, ANOTE LA FECHA DE HOY (EJE.: 24/10/23; EL PRÓXIMO BLOQUE HACIA LA DERECHA SERÁ EL 31/10, ETC.)
5. ANOTE SU PESO SEMANALMENTE POR LOS PRÓXIMOS 6 MESES

NOTA: TABLA EN FONDO BLANCO, PAGINA EN HORIZONTAL

semana	1	2	3	4	5	6	7	8	9	10	11	12	13	14	15	16	17	18	19	20	21	22	23	24
fecha																								

Image S1. Infographic of the pertinent result findings

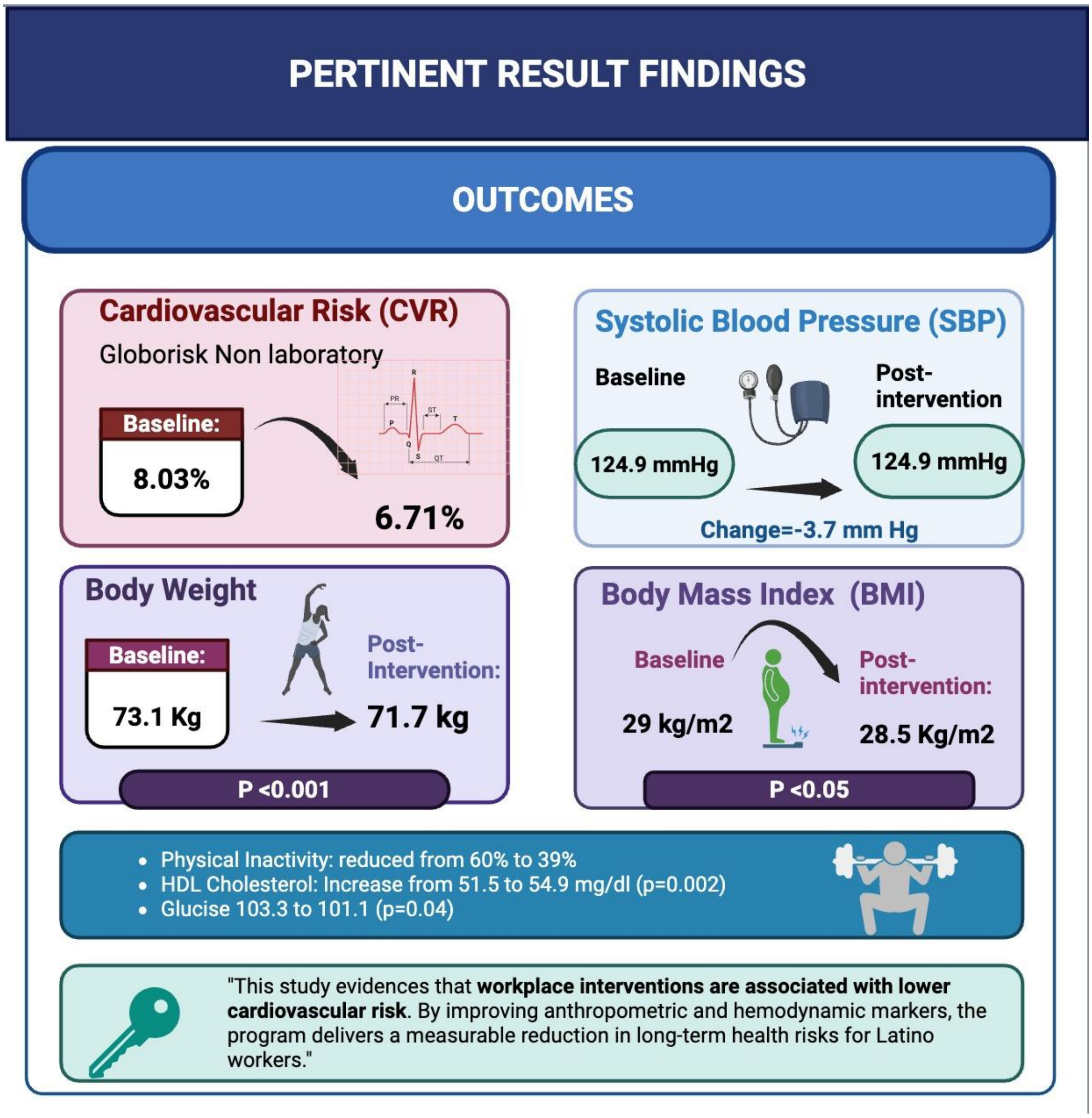

Supplement: Supplementary file 1 [file jcm-15-00628-s001.zip › jcm-4043761-supplementary.pdf]
